# Physiological Functions of Mcl-1: Insights From Genetic Mouse Models

**DOI:** 10.3389/fcell.2021.704547

**Published:** 2021-07-16

**Authors:** Hui San Chin, Nai Yang Fu

**Affiliations:** ^1^Programme in Cancer and Stem Cell Biology, Duke-NUS Medical School, Singapore, Singapore; ^2^Department of Physiology, National University of Singapore, Singapore, Singapore

**Keywords:** Bcl-2, Mcl-1, apoptosis, cell death, mitochondria, genetic mouse model, stem cell

## Abstract

The ability to regulate the survival and death of a cell is paramount throughout the lifespan of a multicellular organism. Apoptosis, a main physiological form of programmed cell death, is regulated by the Bcl-2 family proteins that are either pro-apoptotic or pro-survival. The *in vivo* functions of distinct Bcl-2 family members are largely unmasked by genetically engineered murine models. *Mcl-1* is one of the two Bcl-2 like pro-survival genes whose germline deletion causes embryonic lethality in mice. Its requisite for the survival of a broad range of cell types has been further unraveled by using conditional and inducible deletion murine model systems in different tissues or cell lineages and at distinct developmental stages. Moreover, genetic mouse cancer models have also demonstrated that *Mcl-1* is essential for the survival of multiple tumor types. The *MCL-1* locus is commonly amplified across various cancer types in humans. Small molecule inhibitors with high affinity and specificity to human MCL-1 have been developed and explored for the treatment of certain cancers. To facilitate the pre-clinical studies of MCL-1 in cancer and other diseases, transgenic mouse models over-expressing human *MCL-1* as well as humanized *MCL-1* mouse models have been recently engineered. This review discusses the current advances in understanding the physiological roles of Mcl-1 based on studies using genetic murine models and its critical implications in pathology and treatment of human diseases.

## Introduction

Apoptosis is an evolutionary conserved form of cell death that removes unhealthy and superfluous cells (Kerr et al., 1972). This form of programmed cell death is tightly regulated and is essential for tissue development, homeostasis, and surveillance (Green, 2019; Singh et al., 2019). Consequently, maladaptation in the apoptotic pathway is detrimental, leading to various diseases including autoimmunity (Strasser et al., 1991), degenerative diseases (Bouillet et al., 2001), and cancer (Vaux et al., 1988; Strasser et al., 1990).

**FIGURE 1:**
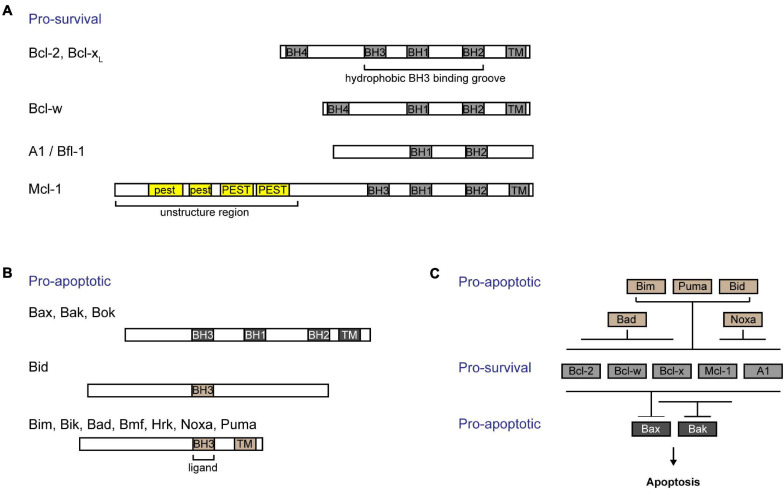
Interactions among Bcl-2 family of proteins confer regulation of the intrinsic apoptotic pathway. Bcl-2 family of proteins are characterized by the presence of Bcl-2 homology (BH) domains. **(A)** Bcl-2 like pro-survival proteins (Bcl-2, Bcl-x_L_, Bcl-w, Mcl-1, and A1/Bfl-1) form a hydrophobic groove that interacts with the BH3 domain of pro-apoptotic proteins. The unique Mcl-1 pro-survival protein is labile and contains a 5’ unstructured region with multiple PEST motifs that are targets for post-translational regulation. **(B)** The pro-apoptotic proteins can be subgrouped into (i) multidomained proteins (Bak, Bax, and Bok) or (ii) BH3-only proteins (Bid, Bim, Bik, Bad, Bmf, Hrk, Noxa, and Puma). **(C)** BH3-only proteins are activated upon stress signals. While Bim, Puma, and Bid are able to bind to all pro-survival proteins, Bad binds specifically to and neutralizes Bcl-2, Bcl-x_L_, and Bcl-w, and Noxa binds to A1/Bfl-1 and Mcl-1. Bcl-x_L_ and Mcl-1 preferentially restrain Bak, while all pro-survival proteins are able to bind to Bax. Importantly, all breaks need to be released from Bax and Bak in order for cells to commit to cell death.

The intrinsic pathway of apoptosis (also known as “Bcl-2 pathway” or “mitochondrial pathway”) is regulated by the Bcl-2 family of proteins. These proteins are characterized by Bcl-2 homology (BH) domains (Czabotar et al., 2014). They can be functionally and structurally organized into three groups: (i) Bcl-2 like pro-survival proteins [Bcl-2 (Vaux et al., 1988), Bcl-x_*L*_ (Boise et al., 1993), Bcl-w (Gibson et al., 1996), Mcl-1 (Kozopas et al., 1993), and A1/Bfl-1 (Lin et al., 1993)] (Figure 1A), (ii) multidomain pro-apoptotic effectors [Bax (Oltvai et al., 1993), Bak (Kiefer et al., 1995), and Bok (Ke et al., 2012)], and (iii) the largely unstructured BH3-only pro-apoptotic initiators [Bim (O’Connor et al., 1998), Bid (Wang et al., 1996), Puma (Han et al., 2001; Nakano and Vousden, 2001), Noxa (Oda et al., 2000), Bad (Yang et al., 1995), Bik (Boyd et al., 1995), Hrk (Inohara et al., 1997), Bmf (Puthalakath et al., 2001), and Moap-1 (Tan et al., 2001; Fu et al., 2007, 2009)] (Figure 1B). Together, they form a tripartite regulatory system that governs the intrinsic apoptotic pathway. The interplay between members of this family of proteins through physical interaction dictates the cell fate, whether to survive or commit cell suicide (Kale et al., 2018; Singh et al., 2019).

B-cell lymphoma 2 (Bcl-2) like pro-survival proteins prevent cell death by sequestering multidomain pro-apoptotic effector proteins Bax/Bak/Bok, thereby preventing downstream cell demise (Willis et al., 2005; Figure 1C). Distinct stress signals activate the apoptotic pathway by engaging different BH3-only proteins either transcriptionally or post-translationally (Adams and Cory, 2007). This in turn either neutralizes Bcl-2 like pro-survival proteins, releasing Bax and Bak, or directly activates Bax and Bak (Letai et al., 2002; Kuwana et al., 2005). Unleashed or activated Bax and Bak subsequently form homo- or hetero-oligomers in the outer mitochondrial membrane (OMM) (Chen et al., 2005; Willis et al., 2007; O’Neill et al., 2016). This compromises the OMM integrity, resulting in the efflux of cytochrome c and other apoptogenic factors from the mitochondrial intermembrane space into the cytosol (Kluck et al., 1997). A cascade of caspase activation is subsequently triggered, which culminates in cell demolition.

*Myeloid cell leukemia-1* (*Mcl-1*) was first identified in 1993 from a screen for genes induced by phorbol 12-myristate 13-acetate in a ML-1 human myeloid leukemia cell line (Kozopas et al., 1993). Mcl-1 protein exhibits significant sequence similarity to Bcl-2 and functions to protect cells from undergoing cell death under various apoptosis-inducing conditions (Kozopas et al., 1993; Reynolds et al., 1994; Zhou et al., 1997). Importantly, the *Mcl-1* locus was shown to be recurrently amplified across multiple tumor types (∼10%) in a large-scale cancer genome study (Beroukhim et al., 2010), and Mcl-1 was found to be highly expressed across a panel of 729 human cancer cell lines (Wei et al., 2012). Subsequently, Mcl-1 was found to be an essential pro-survival molecule in multiple tumor types, warranting the recent advancements of Mcl-1 specific BH3-mimetics in clinical trials, including AMG-176 (Amgen) (Caenepeel et al., 2018), S64315 (Servier) (Szlavik et al., 2020), and AZD5991 (AstraZeneca) (Tron et al., 2018) [reviewed in detail elsewhere (Xiang et al., 2018; Kelly and Strasser, 2020)].

Whole-body deletion of *Mcl-1* in mice results in peri-implantation fatality at E3.5 (Rinkenberger et al., 2000), clearly demonstrating that *Mcl-1* is indispensable for very early embryonic development. Interestingly, the importance of Mcl-1 extends beyond embryonic development. Using conditional genetic mouse model systems, Mcl-1 has been shown to be critical for the survival of various cell types in postnatal mice, including hematopoietic cells, thymic epithelial cells, neurons, cardiomyocytes, hepatocytes, mammary epithelial cells, and reproductive cells. Here, we review the comprehensive genetic mouse model studies on *Mcl-1* in more than two decades and discuss the critical insights and implications generated from these studies.

## Mcl-1, a Unique Bcl-2 Like Pro-Survival Protein

Pro-survival Bcl-2 like proteins contain four BH domains and adopt a similar globular structure with a hydrophobic groove that forms an interface critical for its interactions with other pro-apoptotic proteins. Like other pro-survival Bcl-2 like molecules, Mcl-1 contains a 150 a.a. Bcl-2 like region (i.e., BH1–BH3 domains forming the hydrophobic grove) that interacts with the BH3 domain of pro-apoptotic proteins (Czabotar et al., 2007). Interestingly, Mcl-1 seems to preferably restrain Bak while it also binds to Bax (Willis et al., 2005; Simmons et al., 2008; Figure 1C). Cellular levels of Mcl-1 are regulated transcriptionally, post-transcriptionally, translationally, and post-translationally [reviewed in detail elsewhere (Thomas et al., 2010; Senichkin et al., 2020)]. Different cytokines and growth factors including IL-3 (Wang et al., 1999) and epidermal growth factor (EGF) (Fu et al., 2015; Jain et al., 2018) regulate the expression of *Mcl-1*. Moreover, the abundance of Mcl-1 protein can be tightly controlled through the translational regulation of *Mcl-1* mRNA by the mTOR pathway (Mills et al., 2008; Coloff et al., 2011; Fu et al., 2015). Unlike other pro-survival proteins, the N-terminus of Mcl-1 possesses an additional 170 a.a. extension which contains of multiple PEST motifs (Kozopas et al., 1993). PEST motifs are known to be targeted for protein degradation (Rechsteiner and Rogers, 1996). Indeed, Mcl-1 is a labile protein with a short half-life of ∼30–90 min in most cell types. Accordingly, the basal levels of Mcl-1 protein in cells are regulated in part by the ubiquitin-proteasomal degradation pathway [reviewed in detail elsewhere (Mojsa et al., 2014)]. Currently, there are several known E3 ubiquitin ligases that were found to regulate Mcl-1: Mule (Zhong et al., 2005), βTRcP (Zhong et al., 2005), Fbxw7 (Inuzuka et al., 2011), March5 (Djajawi et al., 2020), and Trim17 (Magiera et al., 2013). Interestingly, the N-terminus of Mcl-1 also contains a mitochondria targeting sequence which directs a truncated form of Mcl-1 to the mitochondrial matrix to promote normal inner mitochondrial membrane structure and mitochondrial respiration (Perciavalle et al., 2012).

## Mcl-1 Haploinsufficiency in Mice

Although complete ablation of *Mcl-1* in mice leads to embryonic lethality, *Mcl-1* haploinsufficiency is well tolerated (Brinkmann et al., 2017). In *Mcl-1*^+/–^ mice, the total body weight, lean mass weight, kidney weight, spleen weight were all reduced when compared to wild type mice. This is more pronounced in males than females, suggesting that males may be more sensitive to the loss of *Mcl-1*. Moreover, the testicular weight of *Mcl-1*^+/–^ male mice was significantly reduced compared to controls. In addition to macroscopic changes, loss of one *Mcl-1* allele led to a reduction in total dendritic cells (DC), particularly in both plasmacytoid DC and conventional DC subpopulations (Carrington et al., 2015). Moreover, the total cellularity of B cells, NK cells and Treg cells were also reduced. To address the requirement of *Mcl-1* in different cell types in postnatal mice, a plethora of conditional knock-out models with specific deletion of *Mcl-1* in different tissues and cell compartments have been developed and extensively studied, which will be summarized and discussed in the following sections.

## Floxed *Mcl-1* Models for Conditional Knockout

**FIGURE 2:**
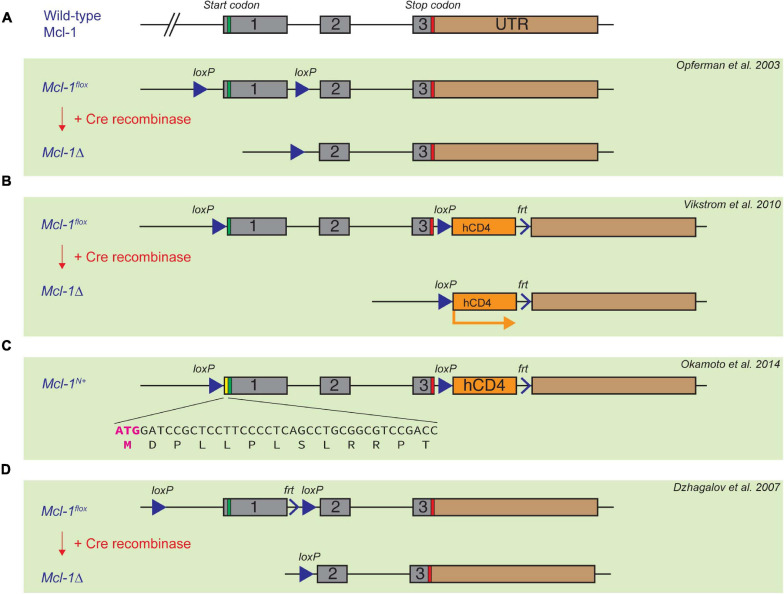
Established floxed *Mcl-1* mouse model systems. **(A)** Opferman et al. introduced two *LoxP* sites to the *Mcl-1* locus upstream of the ATG start codon, and between exon 1 and exon 2, respectively. **(B,C)** In the second floxed *Mcl-1* model, Bouillet et al. introduced two LoxP sites upstream of the ATG start codon of the *Mcl-1* gene, and between exon 3 and 4, respectively. A truncated and non-functional form of hCD4 is introduced downstream of exon 4, which can be subsequently expressed under the control of the endogenous *Mcl-1* promoter upon Cre-induced recombination **(B)**. A serendipitous introduction of 13 extra amino acid in the 5’UTR region of the *Mcl-1* locus resulted in a more stable form of Mcl-1 protein **(C)**. **(D)** Dzhagalov et al. introduced *LoxP* sites flanking exon 1, but the first *LoxP* site is much further upstream of exon 1 in comparison with the other two floxed *Mcl-1* models.

As *Mcl-1* germline deletion causes very early embryonic lethality (Rinkenberger et al., 2000), *Mcl-1* conditional knock-out models become valuable tools to elucidate the functions of Mcl-1 in postnatal mice. Three different floxed *Mcl-1* mouse strains have been reported in literature. In the first floxed *Mcl-1* model generated by Opferman et al. (2003), the *Mcl-1* locus was targeted with *LoxP* sites upstream of the ATG start codon and between exon 1 and exon 2 (Figure 2A). Another floxed *Mcl-1* model was generated by Bouillet et al. (2001), wherein *LoxP* sites were targeted upstream of the ATG start codon and between exon 3 and exon 4 (Vikstrom et al., 2010; Glaser et al., 2012). In this strain, a truncated and non-functional form of hCD4 was also inserted downstream of the *LoxP* sites. Upon Cre recombination, the truncated form of hCD4 is expressed under the control of the endogenous *Mcl-1* promoter (Figure 2B). This serves as a surrogate indicator for successful deletion and also as a reporter for the activity of the *Mcl-1* promoter. Interestingly, homozygous *Mcl-1^*flox/flox*^* males in the above two different floxed *Mcl-1* models were found to be infertile while both males and females did not show overall abnormality over their lifespan. Further studies suggested that the placement of the *LoxP* element at the 5′ UTR in the floxed *Mcl-1* model generated by Bouillet et al. (2001) inadvertently created a new start codon upstream of, and in frame with, the native start codon (Okamoto et al., 2014). This resulted in the production of a stabilized form of Mcl-1 protein with extra 13 a.a. at the N-terminus from the floxed *Mcl-1* allele in the model (Figure 2C). It was initially proposed that the stabilized mutant form of Mcl-1 protein in the model impairs male fertility due to the inhibition of apoptosis, resulting in severe defects in spermatogenesis. However, a recent study indicated that infertility in males could not be rescued when the sequence encoding the additional 13 a.a. in the mutant Mcl-1 protein was removed using CRISPR/Cas9 (Ah-Cann et al., 2016). In the third floxed *Mcl-1* model, *LoxP* sites were placed to flank exon 1 of *Mcl-1* (Dzhagalov et al., 2007; Figure 2D), but the upstream *LoxP* element was integrated into a site with a longer distance from the *Mcl-1* gene, in comparison to the other floxed *Mcl-1* lines. Interestingly, this strategy did not seem to affect male fertility as reported in the other two models. Together, this suggests that minor genomic modification by insertion of a *LoxP* site to the proximal region or 5′ UTR of the murine *Mcl-1* locus can affect spermatogenesis while the underlying mechanism remains unclear. Nevertheless, these various floxed *Mcl-1* knock-out models have been crossed with different *Cre* mouse model systems to delete *Mcl-1* in a cell-type or tissue- specific manner. We summarize the consequence of deleting *Mcl-1* in different tissue using various *Cre*-recombinase mouse model systems below.

## Mcl-1 Is Critical for Multiple Cell Lineages Within the Hematopoietic System

### Hematopoietic Stem Cells and Progenitor Cells

**FIGURE 3:**
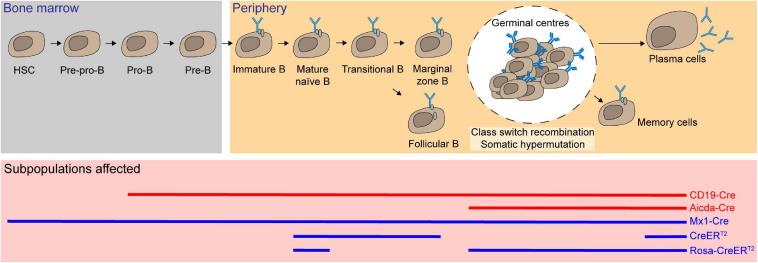
Mcl-1 is an essential pro-survival Bcl-2 protein for hematopoietic stem cells (HSC), progenitor cells, and subsets of B lymphocytes. Using multiple conditional knock-out (red solid line) and inducible conditional knock-out systems (blue solid line), Mcl-1 was found to be crucial for the survival of HSC and all the subsets of B cells, including immature and mature B cells, recirculating B, plasma B, and memory B cells.

By using floxed *Mcl-1* systems, Mcl-1 was found to be essential for multiple cell lineages in the hematopoietic stem cell hierarchy. The *Mx dynamin-like GTPase 1* (*Mx1*) promoter is activated in response to type 1 interferon, which is commonly induced by intraperitoneal administration of polyinosinic-polycytidylic (poly IC) (Kuhn et al., 1995). Ablation of *Mcl-1* in hematopoietic compartment was achieved in the *Mx1-Cre/Mcl-1^*flox/null*^* model (Opferman et al., 2003). This resulted in an overall reduced bone marrow (BM) cellularity with significant depletion of hematopoietic stem cells (HSC) as defined by lineage^–^/cKit^+^/Sca-1^+^ and early hematopoietic progenitors (lineage^–^/cKit^+^/Sca-1^–^). While *Mx1-C*re is able to mediate *Mcl-1* deficiency in multiple lineages, adoptive transfer of BM cells from this model provided evidence suggesting that the essential role of Mcl-1 for maintaining cell survival in HSC and progenitor cells is intrinsic. Moreover, BM progenitors lacking *Mcl-1* were not viable and unable to form colonies in culture. Collectively, these results demonstrate that *Mcl-1* is indispensable for the survival of HSC and hematopoietic progenitor cells (Figure 3).

### B-Cell Lymphocytes

*Mcl-1* transcription levels were shown to decrease as HSC differentiate and commit to either lymphoid or myeloid cell lineages. Nonetheless, *Mcl-1* deletion in BM cells from *Mx1-Cre/Mcl-1^*flox/null*^* mice impaired the growth of HSC, common myeloid progenitor cells and common lymphoid progenitor cells *ex vivo* (Opferman et al., 2005). This suggests that Mcl-1 may also play a critical role in the more differentiated cell types. To interrogate the role of Mcl-1 in B-cell subsets, the *CD19-Cre* transgenic system was employed to achieve *Mcl-1* excision specifically in the B-cell compartment, as early as the pro-B stage (Rickert et al., 1997; Opferman et al., 2003). This resulted in a significant reduction in cellularity in all B-cell subsets. However, this model was unable to discern whether the defect in B-cell compartment is due to the depletion of Mcl-1 in pro-B-cell subpopulation or the survival of subsequent B-cell stages.

To address this, the tamoxifen-inducible *Rosa26-CreER^*T*2^*/*Mcl-1^*flox/flox*^* system was used to efficiently and acutely delete *Mcl-1* in all cell types in adult mice upon tamoxifen administration (Vikström et al., 2016). Although the total cellularity of immature B cells (B220^+^/IgM^+^/IgD^–^) was not significantly reduced when *Mcl-1* deletion was induced, both transitional B cells (B220^+^/IgM^*hi*^/IgD^*int*^) and recirculating mature B cells (B220^+^/IgM^*int*^/IgD^*hi*^) were significantly reduced in the BM. In the spleen, transitional T1 B cells (CD23^–^/IgM^+^/CD21^*lo*^), marginal zone B cells (CD23^–^/IgM^*hi*^/CD21^*hi*^), and mature follicular B cells (CD23^+^/IgM^+^/CD21^+^) were all significantly reduced when *Mcl-1* was deleted. Temporal deletion of *Mcl-1* in B-cell compartment can also be achieved using lethally irradiated BM reconstituted with *Rosa26-CreER^*T*2^/Mcl-1^*flox/flox*^* tamoxifen-inducible cells. Upon acute deletion of *Mcl-1*, total naïve B cells were significantly reduced (Peperzak et al., 2013; Vikström et al., 2016).

B cells undergo extensive proliferation and affinity maturation, and form transient microstructures known as germinal centers (GC) (MacLennan, 1994) where somatic hypermutation and class-switch recombination occur. To examine the role of *Mcl-1* at this stage, the *activation-induced cytidine deaminase*-driven *Cre* (*Aicda-Cre*) transgenic system was crossed with *Mcl-1^*flox/flox*^* mice (Kwon et al., 2008; Vikstrom et al., 2010). The phenotypes observed in *Mcl-1*-deficient mice in this model revealed that GC formation relied on *Mcl-1* in a gene dosage dependent manner. Loss of both alleles of *Mcl-1* resulted in the complete absence of GC formation and the ablation of antigen specific IgG1 B cells in the BM and spleen. GC formation is required for the generation of memory B cells. Accordingly, deletion of *Mcl-1* also resulted in the lack of the memory B-cell population. Significant reduction of serum titers of antigen specific IgG1, but not IgM, suggested that class-switch recombination was severely affected. Notably, loss of one allele of *Mcl-1* resulted in partial loss of these compartments. When the tamoxifen-inducible *Rosa26-CreER^*T*2^*/*Mcl-1^*flox/flox*^* model was utilized to address the function of *Mcl-1* in B-cell regulation after GC formation, it was found that antigen specific B cells and the persistence of GC were both profoundly impaired. Fine-tuned regulation of apoptosis is vital for the selection of high-affinity effector cells. The expression level of Mcl-1 was found to be enhanced via the PI3K signaling pathway in response to the upregulation of cytokine B-cell activating factor (BAFF), which positively correlates with the antigen binding affinity. The B cells with low affinity had limited access to BAFF and were eliminated through apoptosis due to the low expression level of Mcl-1 (Wensveen et al., 2012, 2016). Moreover, in the mice lacking *Noxa*, an BH3-only antagonist of Mcl-1, low-affinity cells persist with increased immunoglobulin diversity and thus mounts suboptimal humoral immune responses (Wensveen et al., 2012).

Most long-lived plasma cells are generated from GC and persist in the BM. These cells can live up to decades in humans. Extracellular cues from their specialized niche are necessary for their longevity. B-cell maturation antigen (BCMA) serves as a receptor for a proliferation-inducing ligand (APRIL) and BAFF that are essential for the survival of the BM plasma cells. It was shown that mRNA and protein expression levels of Mcl-1 were significantly reduced in BM plasma cells in the absence of BCMA (Peperzak et al., 2013). After (4-hydroxy-3-nitrophenyl) acetyl (NP)-keyhole limpet hemocyanin (KLH) immunization, acute deletion of *Mcl-1* using the tamoxifen-inducible *Rosa26-CreER^*T*2^* system led to significant reduction of percentage and cellularity of both total and antigen-specific plasma cells (Peperzak et al., 2013; Vikström et al., 2016). Taken together, *Mc1-1* is critical for multiple stages of B-cell development and for the establishment and maintenance of humoral immunity (Figure 3).

### T-Cell Lymphocytes

Hematopoietic progenitor cells migrate from the BM to the thymus, where thymocytes undergo a series of maturation steps to become T cells. To specifically delete *Mcl-1* in the T-cell compartment, floxed *Mcl-1* mice were crossed with *lymphocyte-specific protein kinase-Cre* (*Lck-Cre*) transgenic mice (Lee et al., 2001; Opferman et al., 2003; Dunkle et al., 2010). In this system, *Mcl-1* was efficiently deleted in the CD4^–^/CD8^–^ double negative (DN) thymocytes. As a result, thymic cellularity was dramatically reduced to only about 5% in comparison to control counterparts, with significant reduction in DN3 (CD44^–^/CD25^+^), DN4 (CD44^–^/CD25^–^), CD4^+^/CD8^+^ double positive (DP), CD4^+^ single positive (SP), CD8^+^ SP cells in the thymus (Opferman et al., 2003). Moreover, the overall cellularity of peripheral T cells was also significantly reduced after *Mcl-1* deletion. A significant proportion of cells were found to undergo apoptosis during the DN2 and DN3 developmental stages in the absence of *Mcl-1*. It is important to note that DN2 cells undergo T-cell receptor rearrangement and are highly dependent upon cytokine signaling for survival. Interestingly, the expression of exogenous Bcl-2 by crossing *Lck-Cre/Mcl-1^*flox/flox*^* mice with *Bcl-2* transgenic mice was unable to rescue the loss of T cells in *Lck-Cre/Mcl-1^*flox*/*flox*^* mice, thus highlighting the unique role of *Mcl-1* in maintaining T-cell survival (Dunkle et al., 2010). To address which of Bak and Bax is important to mediate apoptosis in *Mcl-1*-deficient T cells, *Lck-Cre/Mcl-1^*flox/flox*^* mice were also crossed with *Bak* and *Bax* null mice, respectively (Dunkle et al., 2010). Interestingly, deletion of *Bak*, but not *Bax*, was able to completely restore the DN thymocyte cellularity. However, the absence of *Bax* partially restored the cellularity of thymocytes at subsequent developmental stages, suggesting that *Bax* may also play a role in mediating apoptosis in DP and SP T-cell developmental stage.

To examine the role of *Mcl-1* in later stages of thymocyte development, *CD4-Cre* mice were crossed with floxed *Mcl-1* mice. The total thymic cellularity in the *CD4-Cre/Mcl-1^*flox/flox*^* mice was comparable to that of control mice (Lee et al., 2001; Dzhagalov et al., 2008; Dunkle et al., 2010). In this conditional knockout model, *Mcl-1* was only deleted from the DN4 stage, thereby bypassing the essential requirement for *Mcl-1* in the DN1-DN3 stages. Interestingly, the frequency and cellularity of mature CD4^+^ SP and CD8^+^ SP T cells (TCRβ^+^/Q2^+^/CD69^*lo*^) were substantially reduced in the thymus and periphery of the knockout mice. Notably, the frequency of CD4^+^/CD8^+^/TCRβ^*lo*^ DP cells, which represents a transitional immature stage between DN and DP, was slightly increased in the *Mcl-1*-deficient mice. Importantly, T-cell lymphopenia observed in the *CD4-Cre/Mcl-1^*flox/flox*^* mice was completely rescued by deficiency of *Bak* but not *Bax* (Dunkle et al., 2010). Once again, *Mcl-1* maintains the survival of both CD4^+^ and CD8^+^ T cells by preventing Bak-promoted apoptosis. Additionally, it was shown that pI-pC induced *Mcl-1* excision in *Mx1-Cre/Mcl-1^*flox/null*^* mice resulted in the depletion of T cells in the BM, LN and spleen in a cell intrinsic manner (Opferman et al., 2003). Taken together, *Mcl-1* is not only required for developing T cells, but also for the maintenance of peripheral naïve T cells.

The expression level of Mcl-1 undergoes dynamic changes during T-cell activation. T-cell receptor engagement rapidly induced the upregulation of Mcl-1 via IL-2 signaling (Dzhagalov et al., 2008; Kim et al., 2016). However, 3–5 days after T-cell activation, Mcl-1 protein levels gradually decreased, which was accompanied by a profound induction of Noxa (Chen et al., 2005; Willis et al., 2005; Wensveen et al., 2010). Downregulation and neutralization of Mcl-1 by Noxa in low-affinity T cells also led to the elimination of subdominant clones. Correspondingly, in *Noxa* null mice, Mcl-1 may sustain the survival and expansion of suboptimal T cells with more clonal diversity and low-affinity (Wensveen et al., 2010).

By using the truncated hCD4 as reporter in the floxed *Mcl-1* model (Bouillet et al., 2001), the dynamics of the endogenous *Mcl-1* promoter activity during T-cell development can be monitored. *CD127* (*IL-7R*)-*Cre* transgenic mouse model was widely used to delete a floxed gene in common lymphoid progenitors in the BM and T-cell progenitors in the blood and thymus (Krueger and von Boehmer, 2007; Schlenner et al., 2010). Using the *CD127-Cre/Mcl-1^*flox/flox*^ mouse model*, the highest *Mcl-1* expression was found in the DP stage (Schlenner et al., 2010; Pierson et al., 2013). Interestingly, *Mcl-1* expression was maintained in Foxp3^+^/CD4^+^ T cells while it decreased in conventional CD4^+^ SP T cells (Schlenner et al., 2010). Specific ablation of *Mcl-1* in regulatory T cells by using the *Foxp3-Cre* system resulted in the rapid loss of Foxp3^+^ Treg cells (Gavin et al., 2007; Rubtsov et al., 2008; Pierson et al., 2013). The knockout mice succumbed to fatal immunopathology within ∼4–8 weeks. Moreover, *Foxp3-Cre/Mcl-1^*flox/flox*^* mice exhibited immunological dysregulation, inflammatory infiltrate, hyper-IgE phenotype, elevated mounts of antibodies against dsDNA, abnormally high proliferation of CD8^+^ SP T cells, greater activation of CD4^+^ SP T cells and spontaneous differentiation into TH1, TH2, and TH17 effector cells. These are all the hallmarks of the autoimmune phenotype seen in the *Foxp3*^–^*^/^*^–^ mice (Brunkow et al., 2001; Fontenot et al., 2005).

The function of Mcl-1 in T-cell regulation has also been addressed by challenging the IFN inducible *Mx1-Cre*/*Mcl-1^*flox/null*^* mice with lymphocytic choriomeningitis virus (LCMV). The cellularity of activated CD4^+^ SP and CD8^+^ SP T cells were significantly reduced. This led to a slight reduction in viral load clearance efficiency. Nonetheless, the viral load was completely cleared subsequently due to the persistence of activated T cells that have escape *Mcl-1* deletion. Concomitant loss of both *Bax* and *Bak* were able to rescue the loss of LCMV-specific CD4^+^ SP T cells and CD8^+^ SP T cells (Tripathi et al., 2013). Collectively, data from these studies suggest that Mcl-1 is crucial for survival throughout T-cell development.

### Natural Killer Cells

**FIGURE 4:**
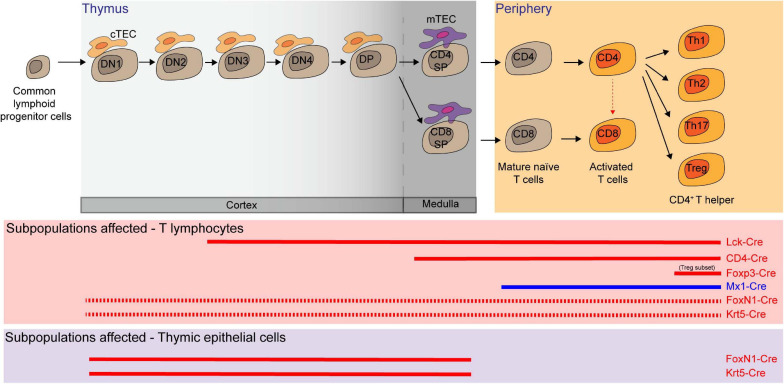
Multiple T lymphocyte lineages and thymic epithelial cells (TEC) rely on Mcl-1 for survival. Conditional knockout (red solid line in the middle panel) and inducible knock-out (blue solid line) models showed that double negative 3 and 4 (DN3 and DN4), double positive (DP), CD4 single positive (SP), CD8 SP, mature T cells, activated T cells, and several subsets of T helper cells are dependent on Mcl-1 for their survival. *FoxN1-Cre* or *Krt5-Cre* mediated *Mcl-1* deletion in TEC (red solid line in the bottom panel) substantially affected the survival of cortical TEC (cTEC) and medullary TEC (mTEC) and led to a significant decrease of all T cell subsets as the consequence of the disruption of thymic microenvironment (red dashed line).

Natural Killer (NK) cells are the most prevalent innate lymphoid cells that are capable of spontaneous cytokine, chemokine, and granulitic production upon activation (Guillerey et al., 2016). The expression of *Mcl-1* is increased in NK cells when they differentiate from immature to mature cells. IL-15 is required for the survival of NK cells and induces the expression of *Mcl-1* mRNA at least partially via Stat5 (Huntington et al., 2007). Consequently, withdrawal of IL-15 leads to the upregulation of Noxa which binds preferentially to Mcl-1 and A1, hence implying that Mcl-1 may be essential for the survival of NK cells (Huntington et al., 2007). When *Mcl-1* was deleted in NK cells using the *Ncr1-Cre* system, both Mature 1 (Mac1^+^/CD27^+^/KLRG1^–^) and Mature 2 (Mac1^+^/CD27^–^/KLRG1^+^) NK cells were completely ablated in all lymphoid organs as well as in the liver (Figure 4; Narni-Mancinelli et al., 2011; Sathe et al., 2014). *Ncr1-Cre/Mcl-1^*flox/flox*^* mice were protected from lethal sepsis when challenged with cecal ligation in puncture, the most common murine model of bacterial sepsis. Due to the NK lymphogenic phenotype, this mouse model may be instrumental to permit further interrogation into the role of *Mcl-1* in NK cells in the context of different diseases. For example, it was found that tumor cells metastasized more easily and extensively when NK cells were absent, but the role of *Mcl-1* in NK cells during tumor initiation and progression remains to be determined.

### Myeloid Cells

To examine the role of Mcl-1 in maintaining the survival of myeloid cells, *Mcl-1^*flox/flox*^* mice were crossed with transgenic mice where the *Cre* expression is driven by the promoter of *Lysozyme M* (*LysM-Cre)* (Clausen et al., 1999) to specifically delete *Mcl-1* in the myeloid compartment including monocytes, macrophages and granulocytes (Dzhagalov et al., 2007; Steimer et al., 2009). *LysM-Cre/Mcl-1^*flox/flox*^* mice showed severe neutropenia due to excessive apoptosis, which was rescued by the co-deletion of both *Bax* and *Bak*, but not either alone (Steimer et al., 2009). Remarkably, neutrophils were ablated, but monocytes, macrophages, and eosinophils were normal in the BM of this conditional *Mcl-1* knockout model (Figure 4; Dzhagalov et al., 2007; Steimer et al., 2009; Csepregi et al., 2018). Moreover, monocytes and macrophages were efficiently recruited to sites of inflammation, whilst mature granulocytes were completely absent. Interestingly, the number of splenic macrophages was significantly increased in *LysM-Cre/Mcl-1^*flox/flox*^* mice while *Mcl-1* deletion rendered macrophages more susceptible to cell death induced by bacterial phagocytosis. This model has been utilized to evaluate the role of neutrophils in various disease contexts. For example, it was found that autoantibody-induced arthritis and anti-CVII antibody-induced dermatitis, which are both known to be dependent on neutrophils, were completely blocked in *LysM-Cre/Mcl-1^*flox/flox*^* mice (Csepregi et al., 2018). When the more neutrophil-specific *Mrp8-Cre* transgenic system was used to delete *Mcl-1* in neutrophils (Figure 4; Passegué et al., 2004; Csepregi et al., 2018), ablation was achieved in up to 99.1% of the neutrophil population. *Mcl-1* deletion in this transgenic model resulted in a reduction in survival, severe wasting phenotype, and compromised breeding productivity.

Other than neutrophils, mast cells and basophils were also shown to rely on *Mcl-1* for survival. *Carboxypeptidase A3-Cre* (*Cpa3-Cre*) mediated *Mcl-1* deletion led to a significant loss of mast cells and basophils but sparing all other myeloid cells (Figure 4; Lilla et al., 2011; Min, 2011). As *Cpa3* is expressed at high levels in mast cells and low levels in basophils, eosinophils, and neutrophils, the *Cpa3-Cre/Mcl-1^*flox/flox*^* model serves as a cKit independent mouse model to uncover the integral role of mast cells in various diseases, including passive cutaneous anaphylaxis, allergen induced skin inflammation, peanut induced anaphylaxis, IgE response to honeybee venom, gram-positive bacteria colonization and infection response, graft versus host disease and osteoarthritis (Lilla et al., 2011; Leveson-Gower et al., 2013; Marichal et al., 2013; Reber et al., 2013; Ando et al., 2015; Gendrin et al., 2015; Wang et al., 2019). To specifically investigate the role of *Mcl-1* in mucosal mast cells, the *Chymase-Cre* (*Chym-Cre*) system has be utilized (Figure 4; Luo et al., 2019). The number of gastric, duodenal mucosal and uterus mast cells was all found to decrease in *Chym-Cre*/*Mcl-1^*flox/flox*^* mice.

### Dendritic Cells

**FIGURE 5:**
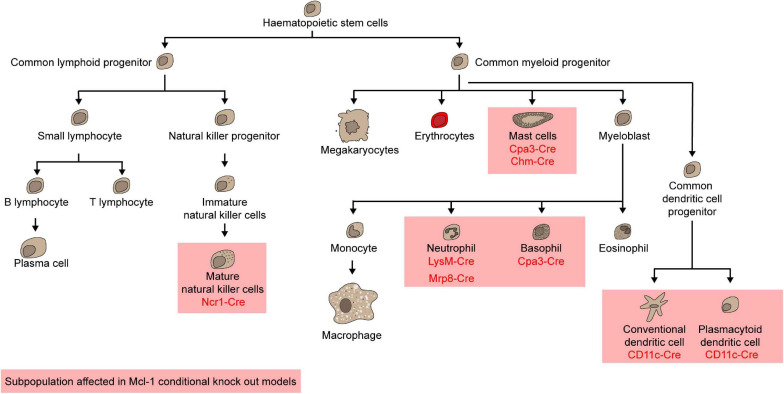
Mcl-1 is indispensable for mature natural killer cells, mast cells, neutrophils, basophils, conventional dendritic cells, and plasmacytoid dendritic cells.

Dendritic cells (DCs) are specialized antigen-presenting cells that are necessary for inducing an effective adaptive immune response. There are broadly two major subsets of DCs: conventional DCs (cDCs) and plasmacytoid DCs (pDCs) (Liu, 2005; Steinman, 2012). Given that *Mcl-1* haploinsufficiency led to a reduction in the total numbers of both cDCs and pDCs (Carrington et al., 2015), it is likely that *Mcl-1* is necessary for the survival of both populations. *Mcl-1* can be deleted restrictedly in DCs using *CD11c-Cre/Mcl-1^*flox/flox*^* mice (Vikstrom et al., 2010; Premsrirut et al., 2011). Deletion of *Mcl-1* led to dramatic reduction in cellularity of pDCs and cDCs in all lymphoid organs in a cell intrinsic manner (Figure 5; Carrington et al., 2015). In these *Mcl-1*-deficient mice, antigen presentation, T-cell priming, and cytokine production were attenuated, suggesting that *Mcl-1* is necessary for the survival of cDCs and pDCs survival and for mounting an effective adaptive immune response.

## The Role of *Mcl-1* in Other Tissues

### Thymic Epithelial Cells

Thymic epithelial cells (TEC) form the specialized niche required for proper T-cell maturation in the thymus (Kumar et al., 2018). Given that *FoxN1* is highly expressed in TEC, *FoxN1-Cre* transgenic mice were used to explore the role of *Mcl-1* in TEC. *FoxN1-Cre* driven *Mcl-1* deletion in mice led to severe thymic atrophy with a substantial decrease in the cellularity of major TEC sub-populations (cTEC, mTEC^*lo*^, AIRE^–^/mTEC^*hi*^, and AIRE^+^/mTEC^*hi*^) (Zuklys et al., 2009; Jain et al., 2018). This impacted the overall thymic architecture, with an extensive presence of ER^–^TR7^+^ thymic fibroblast and progressive disruption of the cortical and medullary regions. The disruption in thymic microenvironment significantly impaired T-cell development, resulting in severe T-cell lymphopenia, with significant reduction in the total cellularity of DN, DP, and SP populations. Importantly, co-deletion of *Bak* alone was able to rescue thymic atrophy, all TEC cellularity and compositional changes and thymic function in the *FoxN1-Cre/Mcl-1^*flox/flox*^* mice (Figure 4). In parallel, when deletion of *Mcl-1* mediated by a *Cre* system where the expression of *Cre* was driven by the promoter of a broad epithelial cell marker Krt5 (*Krt5-Cre)*, similar phenotypes in the thymus were observed (Figure 4). Collectively, these suggest that *Mcl-1* is critical for maintaining the survival of TEC compartment throughout thymus development.

### Mammary Epithelial Cells

Mammary glands undergo extensive proliferation and remodeling during different stages of postnatal development (Fu et al., 2020). Deletion of *Mcl-1* in both luminal and basal epithelial compartments of the mammary gland by either *MMTV-Cre* or *Krt5-Cre* system profoundly delayed the epithelium expansion and ductal tree extension during puberty (Fu et al., 2015). Notably, *Mcl-1*-deficient mammary glands in adult female mice lack mammary stem cells thus impairing alveolar expansion during pregnancy. Consequently, neither *MMTV-Cre/Mcl-1^*flox/flox*^* nor *Krt5-Cre/Mcl-1^*flox/flox*^* dams were able to nurse their pups due to their inability to produce milk, leading to the death of newborn pups within 12–24 h after birth. As expected, excessive apoptosis was readily detected in *Mcl-1*-deficient mammary glands during puberty or pregnancy while proliferation rates of mammary epithelial cells were normal.

At the onset of lactation, Mcl-1 protein expression was dramatically upregulated in the alveolar luminal cells in the mammary gland, which is mediated by the EGF/mTOR signaling axis. However, upon the initiation of involution, the expression level of Mcl-1 protein was rapidly downregulated and the apoptosis cascade was activated in these no longer needed milk-producing cells. The tamoxifen-inducible *Rosa26-CreER^*T*2^* system was used to delete *Mcl-1* at early lactation stage. In this model, mammary glands exhibited signs of involution shortly after *Mcl-1* was acutely deleted. Moreover, deletion of *Mcl-1* specifically in milk-producing cells in lactating mammary glands after the formation of mature alveolar units by using the *WAP-iCre* system resulted in stunted pups with little milk in their stomachs (Wintermantel et al., 2002). In this model, severe premature involution at early lactation was detected. Taken together, *Mcl-1* is the essential member among the Bcl-2 pro-survival proteins required for the survival of mammary epithelial cells across all stages during postnatal development of the mammary gland.

### Liver

*Mcl-1* deletion in the liver epithelium (i.e., hepatocytes and cholangiocytes) using the *Albumin-Cre (Alb-Cre)/Mcl-1^*flox/flox*^* system resulted in spontaneous induction of apoptosis with evidence of liver damage (Postic and Magnuson, 2000; Vick et al., 2009). Interestingly, more than 50% of the *Alb-Cre/Mcl-1^*flox/flox*^* mice developed spontaneous hepatocellular carcinoma around the age of 8–12 months, independent of overt hepatitis (Weber et al., 2010). The underlying mechanism for liver tumorigenesis caused by *Mcl-1* deficiency is likely due to excessive apoptosis in hepatocytes, which is known to induce overwhelming inflammation responses and promote cancer development in the liver (Boege et al., 2017; Hirsova et al., 2017). More recently, *Mcl-1* deficiency in the liver was shown to exacerbate the non-alcoholic steatohepatitis (NASH) phenotype with progression to liver cirrhosis and/or liver tumor in an obesity induced NASH model (Hirsova et al., 2020). In contrast, overexpression of a human *MCL-1* minigene rendered hepatocyte resistant to apoptosis, livery injury and subsequent liver fibrosis induced by bile duct ligation (Zhou et al., 1998; Kahraman et al., 2009).

### Oocytes

During the transition from primordial follicle to primary follicle, Mcl-1 expression increases and accumulates with sustained follicle growth. Moreover, downregulation of Mcl-1 expression precedes oocyte atresia, indicating its role in sustaining the survival of oocytes. To interrogate the requirement of *Mcl-1* in oocytes, *Mcl-1* was efficiently and specifically deleted using the *zona pellucida 3* (*Zp3)-Cre*/*Mcl-1^*flox/flox*^* model (Lewandoski et al., 1997; Opferman et al., 2003; Omari et al., 2015). At the onset of puberty, although the number of oocytes was comparable to the counterparts, females lacking *Mcl-1* showed a significant reduction in growing follicle numbers and the overall ovarian size, likely due to excessive apoptosis in primordial follicles. Consequently, *Zp3-Cre/Mcl-1^*flox/flox*^* females were unable to breed by the age of 4 months. The defects in oocytes caused by *Mcl-1* deficiency could be rescued by co-deleting *Bax*. Together, these data suggest that *Mcl-1* is critical for the survival of growing follicles and hence the maintenance of ovarian reserve.

### Endothelial Cell Survival During Angiogenesis

The importance of Mcl-1 for survival extends beyond its role in the immune system and epithelial cells. The *Tie2-Cre* system has been applied to excise *Mcl-1* specifically in endothelial cells (EC) in multiple studies (Kisanuki et al., 2001; Vikstrom et al., 2010; Watson et al., 2016). Homozygote embryos could only survive up to E15.5 in these models, showing signs of edema, hemorrhage, lack of heartbeat, and embryo reabsorption. *Mcl-1*-deficiency in endothelial cells delayed vascularization in the subcutaneous dorsal skin. Importantly, when both *Bax* and *Bak* were absent in this conditional *Mcl-1* knockout model, pups were born at the mendelian ratio and survived up to 6 weeks of age without overt phenotype. Moreover, the extent of vasculature in the *Mcl-1*-deficient mice was normal when both *Bax* and *Bak* were absent. To overcome embryonic lethality associated with *Tie2-Cre/Mcl-1^*flox/flox*^* mice, the tamoxifen-inducible *Cdh5(PAC)-CreER^*T*2^* system was used to delete *Mcl-1* in endothelial cells in different postnatal stages (Vikstrom et al., 2010; Wang et al., 2010; Watson et al., 2016). Neonatal *Mcl-1* deletion led to a gene-dosage dependent effect on vascular density, with significantly less vessel surface area, segments, and branch points. Moreover, an increased apoptotic rate was observed amongst ECs lacking *Mcl-1* in both the remodeling and sprouting zones, where ECs proliferate and sprout to form new vessels.

### Cardiomyocytes

*Muscle creatine kinase* (*Ckmm*) is expressed in both skeletal and cardiac muscles (Li et al., 2000). *Ckmm-Cre* mediated deletion of *Mcl-1* caused fatality within 10 days post-birth with evidence of rapid and fatal cardiomyopathy, including thinning of the heart walls, cardiac dilation, thrombus deposition, and interstitial fibrosis (Wang et al., 2013). Intriguingly, skeletal muscles were normal in these mice, suggesting that *Mcl-1* is crucial for the survival of cardiac muscle, but not skeletal muscle cells. The cardiac function was improved when both *Bax* and *Bak* were absent in the *Ckmm-Cre/Mcl-1^*flox/flox*^* mice. However, given that *Ckmm* is expressed in cardiac and skeletal muscles at as early as E14, it is uncertain whether the cardiomyopathy phenotypes observed in *Ckmm-Cre/Mcl-1^*flox/flox*^* pups were due to defects during embryonic development. To ascertain the role of *Mcl-1* in cardiac muscles in adult mice, *Mcl-1* was deleted by using the tamoxifen-inducible *Myh6-CreER* mouse model (Sohal et al., 2001; Thomas et al., 2013; Wang et al., 2013). Remarkably, adult mice also showed severe cardiomyopathy, including cardiac dilation, decrease in cardiac wall thickness, cardiac fibrosis and inflammation. Moreover, the majority of knockout mice experienced cardiac failure within 3 weeks. All these phenotypes were rescued by the co-deletion of both *Bax* and *Bak*, suggesting that *Mcl-1* is critical for the survival and function of adult cardiac muscle.

### Nervous System

**FIGURE 6:**
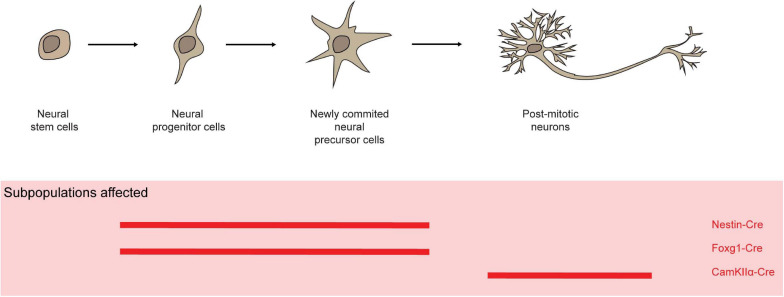
Mcl-1 plays an important role for the survival of neurons and neural progenitor cells. In several conditional knock-out models (red solid line), Mcl-1 was shown to be crucial for the survival of neurons in both the developing and post-mitotic neurons in brain.

*Mcl-1* is highly expressed in proliferating neural precursor cells in the ventricular zone and postmitotic neurons in the developing cortical plate (Arbour et al., 2008). *Nestin* is expressed through the developing nervous system at E7.5 after preplate formation (Dahlstrand et al., 1995). *Nestin-Cre* mediated *Mcl-1* deletion in progenitor cells in the neuroectoderm resulted in embryonic lethality before E15 with evidence of impaired cortex development with elevated levels of apoptosis in neural progenitor cells, newly committed neurons, and migratory neuroblast (Figure 6; Bérubé et al., 2005; Arbour et al., 2008). Similarly, *Foxg1-Cre*-mediated *Mcl-1* deletion in neural progenitors throughout the developing telencephalon caused embryonic lethality at around E16–17 (Hébert and McConnell, 2000; Arbour et al., 2008). The developing brain in homozygote mutant embryos was dramatically smaller in comparison to controls, likely attributed to the increased apoptotic events. Additionally, increased rates of apoptosis were detected in post-mitotic neurons in *CamKIIα-Cre/Mcl-1^*flox/flox*^* mice (Figure 6; Casanova et al., 2001; Germain et al., 2011). Of note, the alpha-isoform of *calcium/calmodulin-dependent protein kinase II* (*CamKII*α) is abundantly expressed in the forebrain and has an essential role in synaptic integrity and plasticity (Arruda-Carvalho et al., 2014). Hence, *Mcl-1* is crucial for the survival of neurons during development as well as post-mitotic neurons in brain.

## Hypomorphic *Mcl-1* Promoter Knock-In Mouse Model

It has been shown that IL-3 promotes the transcription of *Mcl-1* via two promoter elements: sis-inducible element (SIE) and cAMP response elements (CRE) sites (Wang et al., 1999). Targeted mutations in these sites in the mouse model resulted in a hypomorphic phenotype specifically in the thymus, but sparing all other organs (Yang et al., 2009). Whilst the expression of Mcl-1 was reduced in the thymus in this model, Mcl-1 levels remained the same in other organs, suggesting the expression of *Mcl-1* is regulated through different mechanisms in different tissues. Although total thymic cellularity was comparable with the control mice, the number of CD4^–^/CD8^–^, CD4^+^, CD8^+^, CD4^+^/CD8^+^ expressing TCRβ^+^ and CD69^+^, was significantly decreased in mutant mice comparing to the controls. However, T-cell receptor (TCR-α) rearrangement was unaffected.

## Mcl-1 Transgenic Mouse Models

In view of the importance of Mcl-1 in maintaining the survival of multiple cell types, the impacts of overexpression of *Mcl-*1 in transgenic mice were also investigated. Exogenous expression of *Mcl-1* in mice harboring a mini human *MCL-1* transgene and its presumed regulatory elements led to an enhanced viability of hematopoietic cells of various lineages (Zhou et al., 1998). Moreover, transgenic mice had enlarged spleens with fairly normal splenic architecture. Nonetheless, total splenic cellularity of B and T cells was increased in this transgenic model. Immunophenotypic analyses revealed that the proportions of B and T cells were normal without preferential skewing of any subpopulation. When activated, the viability of antigen-specific CD8^+^ SP T cells was enhanced, during acute phase viral infection, memory precursor cell formation, and viral-specific memory T-cell formation (Gui et al., 2015; Kim et al., 2016). Consequently, this led to an increase in the cellularity of antigen-specific CD8^+^ SP T cells upon secondary challenge. Overexpression of Mcl-1 was also predisposed these transgenic mouse model to late-onset of B-cell lymphoma with a spectrum of histological subtypes including follicular lymphoma and diffuse large B-cell lymphoma (Zhou et al., 2001). Importantly, the disease developing in this model was widely disseminated and was of clonal B-cell origin. In line with the role of endogenous Mcl-1 in maintaining the survival of multiple myeloid cell types, expression of exogenous Mcl-1 resulted in the enhanced survival of these myeloid cells. Interestingly, HSC and hematopoietic progenitor cells from the transgenic mice showed enhanced capacity to form lymphoid, myeloid and erythroid colonies *in vitro*.

To clarify the role of *Mcl-1* overexpression in hematopoietic cells, a mouse strain where mouse *Mcl-1* cDNA transgene is driven by promoter/enhancer elements of the *vav* gene was generated (Ogilvy et al., 1999; Campbell et al., 2010). The *vavP-Mcl-1* transgenic mice displayed elevated white blood cell counts, mature B and T lymphocytes, and monocytes. Interestingly, granulocytes, red blood cells and platelet counts were comparable to those of controls. Both splenomegaly and lymphadenopathy were observed in this *Mcl-1* transgenic model. Elevated B and T cells were also evident in these lymphoid organs due to enhanced survival rather than proliferation. Overexpression of *Mcl-1* also provided protection to lymphocytes and granulocytes against cellular stress such as cytokine deprivation and DNA damage agents. Interestingly, there was not any overt phenotype in the BM and thymus in this model. Unlike the *vavP-Bcl-2* model that develops autoimmune kidney disease, the *vavP-Mcl-1* transgenic mice did not develop signs of autoimmunity. Nonetheless, the overexpression of *Mcl-1* exacerbated lpr autoimmune phenotypes and accelerated the morbidity with excessive weight loss, breathing difficulties and severe lymphadenopathy in the *Fas*^*lpr/lpr*^ mouse model (non-functional Fas death receptor) (Cohen and Eisenberg, 1991; Anstee et al., 2017).

## Role of Mcl-1 in Mouse Cancer Models

The *Mcl-1* locus was found to be somatically amplified and its expression found to be elevated in multiple human tumor types and cancer cell lines (Beroukhim et al., 2010; Schwickart et al., 2010; Zack et al., 2013). Indeed, increasing evidence suggests that Mcl-1 is important in melanoma (Sale et al., 2019), hepatocellular carcinoma (Sieghart et al., 2006), breast cancer (Campbell et al., 2018), and various hematological malignancies (Wei et al., 2020). Numerous genetic mouse models have been developed to address the role of Mcl-1 in tumor initiation and progression. In the *vavP-Mcl-1* transgenic mice, overexpression of *Mcl-1* in all hematopoietic compartment predisposes these mice to late onset lymphoma with a phenotype resembling hematopoietic stem cells/progenitor cells expressing both B- and T-cell markers. While some developed pre- B-, B-cell tumors and less frequently, myeloid tumors. The late onset of these tumors suggests that additional mutations are required for malignant transformation in this mouse model. Nevertheless, the tumor types in this model differ from those developed in mice harboring a human *MCL-1* minigene, which displayed tumors resembling follicular lymphoma and diffuse large B-cell lymphoma (Zhou et al., 1998). It is likely that their distinct expression patterns regulated by distinct promoters contributed to the differences in the development of tumor types between the two distinct models.

*Mcl-1* is critical for the survival of rapidly proliferating hematopoietic progenitors and non-transformed pro-B and pre-B cells, which are thought to be the cells of origin in the Eμ-Myc driven lymphoma mouse model (Adams et al., 1985). To test the role of *Mcl-1* in this model, *Mcl-1* was specifically depleted in the B-cell lineage by crossing *E*μ*-Myc* transgenic mice with mice where *Mcl-1* was deleted by the *CD19-Cre* or *Rag-Cre* system (Grabow et al., 2016). *Mcl-1* deletion in the late pro-B-cell stage by the *CD19-Cre* system slightly delayed *E*μ*-Myc* lymphomagenesis. Remarkable, all tumors that arose in *E*μ*-Myc*/*CD19-Cre/Mcl-1^*flox/flox*^* mice retained Mcl-1 expression due to the silencing of *Cre* expression or mutations in the floxed *Mcl-1* allele to escape deletion. Thus, it is likely that there is a selection against *Mcl-1* loss in Eμ-Myc tumors, which suggests Mcl-1 dysregulation is prerequisite for tumor development in this model. The *Rag-Cre* system is commonly used to mediates gene deletion in the common lymphoid progenitor cells. Interestingly, deletion of even one allele of *Mcl-1* by *Rag-Cre* in the *E*μ*-Myc* mouse model led to a significant delay in the onset of lymphomagenesis. In line with this, the overexpression of *Mcl-1* dramatically accelerated the onset of lymphoma developed in the *E*μ*-Myc* tumor model and were resistant to *in vivo* treatment using cyclophosphamide (Adams et al., 1985; Campbell et al., 2010). To address the role of *Mcl-1* in the maintenance of *E*μ*-Myc* driven tumor, *Mcl-1* was deleted by using the tamoxifen-inducible *Rosa26-CreER^*T*2^* system after the tumors have established in mice. Mice with *Mcl-1* deletion displayed improved survival, with 30% of these mice experiencing tumor regression (Kelly et al., 2014). Collectively, this suggest that *Mcl-1* is critical for both the initiation and progression of tumor in *E*μ*-Myc* mice.

*Mcl-1* was also found to be a critical survival protein for other AML mouse models. Lethally irradiated mice transplanted with MLL-ENL oncogene transduced *Rosa26-CreER^*T*2^/Mcl-1^*flox/flox*^* BM cells develop monocytic and myelomonocytic AML in mice. *Mcl-1* deletion resulted in abundant apoptosis in the BM burdened with MLL-ENL induced AML (Lavau et al., 2000; Glaser et al., 2012). This led to the clearance of leukemic blast cells and prolonged survival of mice lacking *Mcl-1*. This strongly suggests that Mcl-1 is a critical pro-survival molecule for the establishment and maintenance of Myc- and MLL-ENL- driven AML. Interestingly, even the loss of one *Mcl-1* allele was sufficient to block the establishment of Myc induced AML (Xiang et al., 2010; Grabow et al., 2016). Furthermore, deletion of a single allele of *Mcl-1* in secondary transplanted Myc-induced AML was also able to significantly prolong the survival of secondary recipient mice. Importantly, transcriptional profiling showed that MCL-1 expression is consistently elevated in primary human AML samples (Kaufmann et al., 1998).

In addition, the loss of a single *Mcl-1* allele was sufficient to delay the onset of and reduce the incidences of thymic T-cell lymphoma driven by the absence of *p53* (Grabow et al., 2014). Similarly, loss of a single *Mcl-1* allele also delayed tumor onset and impaired the survival of T-cell lymphomas developed in a T lymphocyte non-Hodgkin’s lymphoma (T-NHL) mouse model driven by the fusion kinase ITK-SYK signaling. Moreover, it was found that *Mcl-1* was highly expressed in tumor lesions in a genetic mouse model of breast cancer, *MMTV-PyMT*. Importantly, there was a strong selection against the loss of *Mcl-1* when this model was crossed with *MMTV-Cre/Mcl-1^*flox/flox*^* model (Campbell et al., 2018). Together, these data highlight the integral role of *Mcl-1* not only for maintaining the survival of established malignancies, but also for driving tumorigenesis (Spinner et al., 2016). It is plausible to speculate that many of the malignancies with elevated expression of Mcl-1 will benefit from treatment with Mcl-1 specific inhibitors either alone or in combination with other anti-cancer agents (Merino et al., 2018; Xiang et al., 2018; Kelly and Strasser, 2020).

## Humanized Mcl-1 Mouse Models

BH3-mimetics are a class of small molecules that initiate the apoptotic pathway by mimicking the action of BH3-only proteins, the natural inhibitors of Bcl-2 pro-survival proteins. Given the remarkable clinical success of the Bcl-2 specific inhibitor, venetoclax or venclaxta, in treating relapsed or refractory chronic lymphocytic leukemia (CLL), many have sought to develop a Mcl-1 specific inhibitor. Recently, several small molecule Mcl-1 specific inhibitors, S63945 and S64315 (Servier), AMG-176 (Amgen), and AZD5991 (AstraZeneca) have been developed and shown to be efficacious in a broad panel of cell lines *in vitro* and in xenograft models. Interestingly, these compounds were found to bind to human MCL-1 (*huMCL-1*) with considerably higher affinity than mouse Mcl-1 (6–1000 folds for different inhibitors). Thus, to model the efficacies and determine the on-target toxicity of these agents with more accuracy, humanized *MCL-1* mouse models were generated by two independent groups. In these mouse models, the native mouse *Mcl-1* locus was replaced with human homolog, maintaining the flanking 5′ and 3′ UTR (Brennan et al., 2018; Caenepeel et al., 2018). Phenotypically, the humanized *Mcl-1* mouse models were indistinguishable to wild-type mice under normal physiological condition. The proportions and numbers of lymphoid cells, myeloid cells were comparable between *huMCL-1* mouse models and wild-type controls. huMCL-1 is able to bind to murine Bak and Bax and the overall apoptotic machinery remains largely intact in these models. Thus, these models would serve as useful tools for pre-clinical validation and to determine the efficacies and tolerability of Mcl-1 inhibitors for treating cancer and other diseases (Kotschy et al., 2016).

## Non-Apoptotic Roles of Mcl-1

Although the canonical role of Mcl-1 is to promote cell survival by neutralizing multidomain proapoptotic protein Bax/Bak, non-apoptotic roles have recently been attributed to Mcl-1 [reviewed in detail elsewhere (Perciavalle and Opferman, 2014)]. Two distinct forms of Mcl-1 protein have been shown to reside in different locations of the mitochondria (OMM and matrix) and may contribute differently to the proper mitochondria function. Whilst Mcl-1 in the OMM seem to only interact with the Bcl-2 family proteins, Mcl-1 in the matrix contributes to mitochondrial fusion, ATP production, mitochondrial membrane potential and structure, mitochondrial respiration and maintaining oligomeric ATP synthase (Perciavalle et al., 2012). Interestingly, the deletion of *Mcl-1* in cardiac muscles caused mitochondrial abnormalities including disrupted myocardium with disorganized mitochondria with abnormal cristae structure (Thomas et al., 2013; Wang et al., 2013). Electron microscopy showed that the cardiac mitochondria were swollen at baseline and exhibited modest calcium-induced swelling. Whilst the co-deletion of both *Bax* and *Bak* rescued the cardiomyopathy associated with the loss of *Mcl-1*, it did not reverse the mitochondrial abnormalities in cardiac muscles. Similarly, mitochondrial dysfunction associated with *Mcl-1*-deficiency in oocytes was not rescued by *Bax* deletion (Omari et al., 2015).

In addition, the isoform of Mcl-1 present in the mitochondria matrix was found to interact with very long-chain acyl-CoA dehydrogenase (VLCAD) (Escudero et al., 2018), which is important for catalyzing fatty acid β-oxidation, converting energy stored in fats into ATP. Mice lacking *Mcl-1* in the liver were reconstituted with either the OMM isoform (Mcl-1^*OMM*^) or matrix isoform (Mcl-1^*matrix*^) and subjected to murine liver proteomics analysis (Perciavalle et al., 2012; Escudero et al., 2018). Whilst Mcl-1^*OMM*^ expectedly interacted with members of the Bcl-2 family (Bim and Puma), there was a selective enrichment of VCLAD in the Mcl-1^*matrix*^ pull down (Escudero et al., 2018). As the BH3 domain of Mcl-1 was found to modulate the enzymatic activity of VLCAD, the absence of the Mcl-1^*matrix*^ resulted in a hyperactive fatty acid β-oxidation, which may cause substrate overconsumption and/or co-factor depletion.

Interestingly, Mcl-1 protein has also been found to localize in the nucleus and affect cell cycle progression. Mechanistically, physical interaction of Mcl-1 with the important cell cycle regulator, proliferating cell nuclear antigen (PCNA), may contribute to its inhibition of cell cycle progression through S-phase (Fujise et al., 2000). Moreover, a shortened nuclear form of Mcl-1 protein was also found in to interact with and dampen the kinase activity of cyclin-dependent kinase 1 (Cdk1), thus reducing the proliferation rate in cells (Jamil et al., 2005). Additionally, Mcl-1 was also found to have a role in ATR (AT mutated and Rad3 related)- dependent DNA damage response, by regulating the phosphorylation and activation of DNA damage checkpoint kinase, Chk1 (Jamil et al., 2005). When Mcl-1 is knock-down, Chk1 phosphorylation in response to DNA damage was completely blocked. All together, these studies suggest that Mcl-1 may not only function as an anti-apoptotic Bcl-2 molecule but also play a role in mitochondrial activity, proliferation and DNA damage response.

## Non-Redundancy in the Physiological Function of Pro-Survival Bcl2 Members

Unlike the effector Bcl-2 members Bax/Bak, studies of genetic mouse models suggest that the physiological functions of different pro-survival Bcl-2 members are not redundant in many contexts. Although the expression of different pro-survival proteins can be detected within the same cell type, certain members may play a more dominant role over others during early development as well as later tissue homeostasis and regeneration. Deletion of *Mcl-1* and *Bcl-x* causes embryonic lethality at E3.5 and E13.5, respectively. In contrast, *Bcl-2*, *Bcl-w*, and *A1* deficient mice are able to complete embryonic development. Apart from its critical role in spermatogenesis, the pro-survival function of Bcl-w is otherwise dispensable in most cell types (Print et al., 1998; Ross et al., 1998). Whilst *A1* is largely expressed in the hematopoietic system, complete loss of A1 is well tolerated with only minor defects in unconventional TCRγδ T cells, regulatory T cells, memory CD4^+^ T cells, and conventional dendritic cells (Schenk et al., 2017). Although normal at birth, however, *Bcl-2* null mice showed growth retardation and hypopigmentation due to the loss of melanocytes. Moreover, early mortality was observed amongst a significant proportion of *Bcl-2*-deficient pups, mainly due to polycystic kidney disease as a consequence of excessive apoptosis in the kidney epithelium (Veis et al., 1993). While all hematopoietic lineages in the *Bcl-2*-deficient mice were comparable to control counterparts at birth, the percentage and absolute number of lymphocytes decreased along with significant thymic and splenic atrophy in aging *Bcl-2*-deficient mice, suggesting that *Bcl-2* is required for the maintenance of the lymphoid compartment, but not for hematopoietic cell maturation (Nakayama et al., 1993; Veis et al., 1993). Interestingly, mouse models with *Bcl-2* deficiency or hypomorphism in NK cells revealed that *Bcl-2* is important for the survival of resting NK cells, but it becomes dispensable in cycling NK cells which can reply on *Mcl-1* for survival (Viant et al., 2017).

As described in the sections above, the physiological roles of Mcl-1 have been explored extensively in numerous conditional knockout models. Conditional knock-out mouse models have also been employed to address the *in vivo* role of *Bcl-2* and *Bcl-x* in postnatal mice under the physiological condition (Wagner et al., 2000; Thorp et al., 2009). Remarkably, while *Mcl-1* is crucial for multiple B-cell subsets, *Bcl-x* deficiency only impacted immature B cells sparing all other B-cell subsets (Vikström et al., 2016). In activated B cells, *Bcl-x* was also found to be dispensable for GC formation, memory B cells, antigen-specific B-cell expansion, and plasma cells (Vikstrom et al., 2010; Peperzak et al., 2013). During T-cell development, cells displayed a dynamic expression of Bcl-2 and Bcl-x_*L*_ protein. Whilst Bcl-x_*L*_ was upregulated in DP cells but downregulated in SP cells, Bcl-2 was downregulated in DP cells but upregulated in SP positive cells (Gratiot-Deans et al., 1993, 1994; Grillot et al., 1995). As expected, deficiency of *Bcl-x* led to a decrease in DP population (Zhang and He, 2005; Dzhagalov et al., 2008). In contrast, *Mcl-1* plays a crucial role throughout T-cell development as reviewed in a previous section. While the loss of *Mcl-1* severely affected the survival of thymic epithelial cells and hence, T-cell development, *Bcl-2* and *Bcl-x* were found to be dispensable for these cells (Jain et al., 2018). In the mammary gland, the tissue function and structure are normal in *Bcl-x*-deficient mice across different developmental stages, which is in a striking contrast to the indispensable role of Mcl-1 in the mammary epithelium (Walton et al., 2001; Fu et al., 2015). Although Mcl-1 plays non-redundant roles in various tissues, Bcl-x_*L*_ is the key pro-survival Bcl-2 member for the survival of mature megakaryocytes and platelets in postnatal mice (Mason et al., 2007; Josefsson et al., 2011; Debrincat et al., 2012, 2015). In line with this, pharmacological inhibition of Bcl-x_*L*_ by Navitoclax in adult mice caused on-target thrombocytopenia (Roberts et al., 2012). Notably, the compound deletion of both *Mcl-1* and *Bcl-x* resulted in preweaning lethality due to severely compromised megakaryocyte development, suggesting a redundant role of *Mcl-1* and *Bcl-x* during early megakaryocyte development (Debrincat et al., 2012).

## Conclusion

Mcl-1 has long been recognized as a unique molecule among the pro-survival Bcl-2 family members (i.e., Bcl-2, Bcl-xl, A1, Bcl-w, and Mcl-1) for its molecular features, including its tight control at transcription levels, distinct primary protein sequence, short half-life, special interaction profile with the BH3-only molecules. Extensive genetic mouse model studies in the past more than two decades have clearly shown that *Mcl-1* plays non-redundant and essential physiological roles in the survival of a wide variety of cell types, including stem cells, progenitor cells and fully differentially cells, in many different tissues even when other pro-survival Bcl-2 family members are highly expressed in the same cells. However, the underlying molecular mechanisms for the dominant role of Mcl-1 over other pro-survival Bcl-2 members unraveled by mouse models remain largely unclear. It has been proposed that, compared to other Bcl-2 pro-survival members, Mcl-1 seems to preferably sequester the function of Bak, but not Bax. But whether the phenotypes caused by *Mcl-1* deletion in different cellular contexts are due to the dysregulation of either Bax or Bak alone, or both remains obscure. While certain BH3-only molecules, such as Noxa, may only interact with Mcl-1, but not other pro-survival Bcl-2 family member, the contribution of such BH3-only molecules to the non-redundant role of Mcl-1 in distinct cellular contexts also needs to be investigated further by using new genetic mouse models. Further elucidation of the key up- and down-stream regulators of Mcl-1 function may lead to new insights into alternative and more specific strategies for manipulation of the Mcl-1 pro-survival activity in distinct defined cellular contexts. Non-apoptotic functions of Mcl-1 have been reported recently. Most of those studies, however, were mainly based on *in vitro* cell culture systems. Feasible mouse models need to be developed in future to formally address the importance of these functions under physiological and pathological conditions. Moreover, given the important role of Mcl-1 in cancer, Mcl-1 is regarded as a valid therapeutic target for treatment of certain cancer types, and several inhibitors with high binding affinity and specificity for human MCL-1 protein have been recently developed and showed promising effects in blocking tumor development in mouse models. However, the toxicity of these inhibitors needs to be evaluated carefully in appropriate pre-clinical mouse models expressing human MCL-1 and in patients as Mcl-1 is an essential pro-survival Bcl-2 member in many tissues and cell types under physiological condition. Fortunately, multiple genetic studies provide convincing evidence that knockout of one allele of *Mcl-1* is sufficient to block tumor development, but did not cause severe overall phenotypes in mice, implying the possibility that partial inhibition of Mcl-1 function by specific inhibitors at a right range of dosage could be an effective way for cancer treatment without significant side effect. Notably, the current literature on the role Mcl-1 in cancer mainly focuses on tumor cells. The potential function of Mcl-1 in the establishment and maintenance of tumor microenvironment, including tumor-associated immune cells and fibroblasts, for tumor initiation and progression in different cancer types would be an interesting avenue for future research.

## Author Contributions

HSC and NYF reviewed the literature and wrote the review together. HSC designed and prepared the figures.

## Conflict of Interest

The authors declare that the research was conducted in the absence of any commercial or financial relationships that could be construed as a potential conflict of interest.

## Abbreviations

a.a.: Amino acids
A1: Bcl-2-related protein A1
AML: Acute myeloid leukemia
Bad: Bcl-2 associated agonist of cell death
Bak: Bcl-2 antagonist killer
Bax: Bcl-2 associated X
Bcl-2: B-cell lymphoma 2
Bcl-w: Bcl-2-like protein 2
Bcl-x_*L*_: B-cell lymphoma extra large
Bfl-1: Bcl-2 related protein A1
BH: Bcl-2 homology domain
Bid: BH3 interacting domain death protein
Bik: Bcl-2-interacting killer
Bim: Bcl-2 interacting mediator of cell death
BM: Bone marrow
Bmf: Bcl-2 modifying factor
Bok: Bcl-2 related ovarian killer
cDC: Conventional dendritic cells
cTEC: Cortical thymic epithelial cells
DC: Dendritic cells
DN: Double negative
DP: Double positive
EGF: Epidermal growth factor
GC: Germinal centers
Lck: Lymphocyte-specific protein kinase
hCD4: Human CD4
Hrk: Harakiri
HSC: Hematopoietic stem cells
LN: Lymph node
Mcl-1: Myeloid cell leukemia sequence 1
MLL-ENL: Mixed-lineage leukemia – eleven nineteen leukemia fusion protein
Moap-1: Modulator of apoptosis 1
MOM: Mitochondria outer membrane
mTEC: Medullary thymic epithelial cells
Mx1: Mx dynamin-like GTPase 1
NK: Natural killer cells
pDC: Plasmacytoid dendritic cells
PEST: proline, glutamic acid, serine, theonine
poly IC: Polyinosinic-polycytidylic
Puma: p53 upregulated modulator of apoptosis
SP: Single positive
TCR: T-cell receptor
TEC: Thymic epithelial cells
Treg: T regulatory cells.

## References

[B1] Adams J. M. Cory S. (2007). The Bcl-2 apoptotic switch in cancer development and therapy. *Oncogene* 26 1324–1337. 10.1038/sj.onc.1210220 17322918 PMC2930981

[B2] Adams J. M. Harris A. W. Pinkert C. A. Corcoran L. M. Alexander W. S. Cory S. (1985). The c-myc oncogene driven by immunoglobulin enhancers induces lymphoid malignancy in transgenic mice. *Nature* 318 533–538. 10.1038/318533a0 3906410

[B3] Ah-Cann C. Tailler M. Kueh A. J. Herold M. J. Opferman J. T. Asselin-Labat M. L. (2016). Male sterility in Mcl-1-flox mice is not due to enhanced Mcl1 protein stability. *Cell Death Dis.* 7 e2490–e2492. 27906183 10.1038/cddis.2016.391 PMC5261018

[B4] Ando T. Matsumoto K. Namiranian S. Yamashita H. Glatthorn H. Kimura M. (2015). Mast cells are required for full expression of allergen/SEB-induced skin inflammation. *J. Invest. Dermatol.* 135:925. 10.1038/jid.2014.359 25209389

[B5] Anstee N. S. Vandenberg C. J. Campbell K. J. Hughes P. D. O’Reilly L. A. Cory S. (2017). Overexpression of Mcl-1 exacerbates lymphocyte accumulation and autoimmune kidney disease in lpr mice. *Cell Death Diff.* 24 397–408. 10.1038/cdd.2016.125 27813531 PMC5344201

[B6] Arbour N. Vanderluit J. L. Le Grand J. N. Jahani-Asl A. Ruzhynsky V. A. Cheung E. C. C. (2008). Mcl-1 is a key regulator of apoptosis during CNS development and after DNA damage. *J. Neurosci.* 28 6068–6078. 10.1523/jneurosci.4940-07.2008 18550749 PMC2681190

[B7] Arruda-Carvalho M. Restivo L. Guskjolen A. Epp J. R. Elgersma Y. Josselyn S. A. (2014). Conditional deletion of α-CaMKII impairs integration of adult-generated granule cells into dentate gyrus circuits and hippocampus-dependent learning. *J. Neurosci.* 34 11919–11928. 10.1523/jneurosci.0652-14.2014 25186740 PMC6608460

[B8] Beroukhim R. Mermel C. H. Porter D. Wei G. Raychaudhuri S. Donovan J. (2010). The landscape of somatic copy-number alteration across human cancers. *Nature* 463 899–905. 20164920 10.1038/nature08822 PMC2826709

[B9] Bérubé N. G. Mangelsdorf M. Jagla M. Vanderluit J. Garrick D. Gibbons R. J. (2005). The chromatin-remodeling protein ATRX is critical for neuronal survival during corticogenesis. *J. Clin. Invest.* 115 258–267. 10.1172/jci200522329 15668733 PMC544602

[B10] Boege Y. Malehmir M. Healy M. E. Bettermann K. Lorentzen A. Vucur M. (2017). A dual role of caspase-8 in triggering and sensing proliferation-associated dna damage, a key determinant of liver cancer development. *Cancer Cell* 32 342–359.e310. 28898696 10.1016/j.ccell.2017.08.010 PMC5598544

[B11] Boise L. H. González-García M. Postema C. E. Ding L. Lindsten T. Turka L. A. (1993). bcl-x, a bcl-2-related gene that functions as a dominant regulator of apoptotic cell death. *Cell* 74 597–608. 10.1016/0092-8674(93)90508-n 8358789

[B12] Bouillet P. Cory S. Zhang L. C. Strasser A. Adams J. M. (2001). Degenerative disorders caused by Bcl-2 deficiency prevented by loss of its BH3-only antagonist Bim. *Dev. Cell* 1 645–653. 10.1016/s1534-5807(01)00083-1 11709185

[B13] Boyd J. M. Gallo G. J. Elangovan B. Houghton A. B. Malstrom S. Avery B. J. (1995). Bik, a novel death-inducing protein shares a distinct sequence motif with Bcl-2 family proteins and interacts with viral and cellular survival-promoting proteins. *Oncogene* 11 1921–1928. 7478623

[B14] Brennan M. S. Chang C. Tai L. Lessene G. Strasser A. Dewson G. (2018). Humanized Mcl-1 mice enable accurate preclinical evaluation of MCL-1 inhibitors destined for clinical use. *Blood* 132 1573–1583. 10.1182/blood-2018-06-859405 30139826

[B15] Brinkmann K. Grabow S. Hyland C. D. Teh C. E. Alexander W. S. Herold M. J. (2017). The combination of reduced MCL-1 and standard chemotherapeutics is tolerable in mice. *Cell Death Differ.* 24 2032–2043. 10.1038/cdd.2017.125 28800129 PMC5686343

[B16] Brunkow M. E. Jeffery E. W. Hjerrild K. A. Paeper B. Clark L. B. Yasayko S. A. (2001). Disruption of a new forkhead/winged-helix protein, scurfin, results in the fatal lymphoproliferative disorder of the scurfy mouse. *Nat. Genet.* 27 68–73. 10.1038/83784 11138001

[B17] Caenepeel S. Brown S. P. Belmontes B. Moody G. Keegan K. S. Chui D. (2018). AMG 176, a selective MCL1 inhibitor, is effective in hematologic cancer models alone and in combination with established therapies. *Cancer Discov.* 8 1582–1597. 30254093 10.1158/2159-8290.CD-18-0387

[B18] Campbell K. J. Bath M. L. Turner M. L. Vandenberg C. J. Bouillet P. Metcalf D. (2010). Elevated Mcl-1 perturbs lymphopoiesis, promotes transformation of hematopoietic stem/progenitor cells, and enhances drug resistance. *Blood* 116 3197–3207. 10.1182/blood-2010-04-281071 20631380 PMC2995351

[B19] Campbell K. J. Dhayade S. Ferrari N. Sims A. H. Johnson E. Mason S. M. (2018). MCL-1 is a prognostic indicator and drug target in breast cancer. *Cell Death Dis.* 9:19. 10.1038/s41419-017-0035-2 PMC5833338 29339815

[B20] Carrington E. M. Zhang J. G. Sutherland R. M. Vikstrom I. B. Brady J. L. Soo P. (2015). Prosurvival Bcl-2 family members reveal a distinct apoptotic identity between conventional and plasmacytoid dendritic cells. *Proc. Natl. Acad. Sci. U.S.A.* 112 4044–4049. 10.1073/pnas.1417620112 25775525 PMC4386329

[B21] Casanova E. Fehsenfeld S. Mantamadiotis T. Lemberger T. Greiner E. Stewart A. F. (2001). A CamKIIalpha iCre BAC allows brain-specific gene inactivation. *Genesis (New York, NY : 2000)* 31 37–42. 10.1002/gene.1078 11668676

[B22] Chen L. Willis S. N. Wei A. Smith B. J. Fletcher J. I. Hinds M. G. (2005). Differential targeting of prosurvival Bcl-2 proteins by their BH3-only ligands allows complementary apoptotic function. *Mol. Cell* 17 393–403. 10.1016/j.molcel.2004.12.030 15694340

[B23] Clausen B. E. Burkhardt C. Reith W. Renkawitz R. Forster I. (1999). Conditional gene targeting in macrophages and granulocytes using LysMcre mice. *Transgenic Res.* 8 265–277. 10621974 10.1023/a:1008942828960

[B24] Cohen P. L. Eisenberg R. A. (1991). Lpr and gld: single gene models of systemic autoimmunity and lymphoproliferative disease. *Ann. Rev. Immunol.* 9 243–269. 10.1146/annurev.iy.09.040191.001331 1910678

[B25] Coloff J. L. Macintyre A. N. Nichols A. G. Liu T. Gallo C. A. Plas D. R. (2011). Akt-dependent glucose metabolism promotes Mcl-1 synthesis to maintain cell survival and resistance to Bcl-2 inhibition. *Cancer Res.* 71 5204–5213. 10.1158/0008-5472.can-10-4531 21670080 PMC3148426

[B26] Csepregi J. Z. Orosz A. Zajta E. Kasa O. Nemeth T. Simon E. (2018). Myeloid-specific deletion of Mcl-1 yields severely neutropenic mice that survive and breed in homozygous form. *J. Immunol.* 201 3793–3803. 10.4049/jimmunol.1701803 30464050 PMC6287103

[B27] Czabotar P. E. Lee E. F. Van Delft M. F. Day C. L. Smith B. J. Huang D. C. S. (2007). Structural insights into the degradation of Mcl-1 induced by BH3 domains. *Proc. Natl. Acad. Sci. U.S.A.* 104 6217–6222. 10.1073/pnas.0701297104 17389404 PMC1851040

[B28] Czabotar P. E. Lessene G. Strasser A. Adams J. M. (2014). Control of apoptosis by the BCL-2 protein family: implications for physiology and therapy. *Nat. Rev. Mol. Cell Biol.* 15 49–63. 10.1038/nrm3722 24355989

[B29] Dahlstrand J. Lardelli M. Lendahl U. (1995). Nestin mRNA expression correlates with the central nervous system progenitor cell state in many, but not all, regions of developing central nervous system. *Brain Res. Dev.* 84 109–129. 10.1016/0165-3806(94)00162-s 7720210

[B30] Debrincat M. A. Josefsson E. C. James C. Henley K. J. Ellis S. Lebois M. (2012). Mcl-1 and Bcl-x(L) coordinately regulate megakaryocyte survival. *Blood* 119 5850–5858. 10.1182/blood-2011-12-398834 22374700

[B31] Debrincat M. A. Pleines I. Lebois M. Lane R. M. Holmes M. L. Corbin J. (2015). BCL-2 is dispensable for thrombopoiesis and platelet survival. *Cell Death Dis.* 6:e1721. 10.1038/cddis.2015.97 25880088 PMC4650559

[B32] Djajawi T. M. Liu L. Gong J. N. Huang A. S. Luo M. J. Xu Z. (2020). MARCH5 requires MTCH2 to coordinate proteasomal turnover of the MCL1:NOXA complex. *Cell Death Diff.* 27 2484–2499. 10.1038/s41418-020-0517-0 32094511 PMC7370232

[B33] Dunkle A. Dzhagalov I. He Y. W. (2010). Mcl-1 promotes survival of thymocytes by inhibition of Bak in a pathway separate from Bcl-2. *Cell Death Differ.* 17 994–1002. 10.1038/cdd.2009.201 20057504 PMC2866813

[B34] Dzhagalov I. Dunkle A. He Y. W. (2008). The anti-apoptotic Bcl-2 family member Mcl-1 promotes T lymphocyte survival at multiple stages. *J. Immunol.* 181 521–528. 10.4049/jimmunol.181.1.521 18566418 PMC2561902

[B35] Dzhagalov I. St. John A. He Y. W. (2007). The antiapoptotic protein Mcl-1 is essential for the survival of neutrophils but not macrophages. *Blood* 109 1620–1626. 10.1182/blood-2006-03-013771 17062731 PMC1794052

[B36] Escudero S. Zaganjor E. Lee S. Mill C. P. Morgan A. M. Crawford E. B. (2018). Dynamic Regulation of Long-Chain Fatty Acid Oxidation by a Noncanonical Interaction between the MCL-1 BH3 Helix and VLCAD. *Mol. Cell* 69 729–743.e727. 29499131 10.1016/j.molcel.2018.02.005 PMC5916823

[B37] Fontenot J. D. Rasmussen J. P. Williams L. M. Dooley J. L. Farr A. G. Rudensky A. Y. (2005). Regulatory T cell lineage specification by the forkhead transcription factor Foxp3. *Immunity* 22 329–341. 10.1016/j.immuni.2005.01.016 15780990

[B38] Fu N. Y. Nolan E. Lindeman G. J. Visvader J. E. (2020). Stem cells and the differentiation hierarchy in mammary gland development. *Physiol. Rev.* 100 489–523. 10.1152/physrev.00040.2018 31539305

[B39] Fu N. Y. Rios A. C. Pal B. Soetanto R. Lun A. T. L. Liu K. (2015). EGF-mediated induction of Mcl-1 at the switch to lactation is essential for alveolar cell survival. *Nat. Cell Biol.* 17 365–375. 10.1038/ncb3117 25730472

[B40] Fu N. Y. Sukumaran S. K. Kerk S. Y. Yu V. C. (2009). Baxbeta: a constitutively active human Bax isoform that is under tight regulatory control by the proteasomal degradation mechanism. *Mol. Cell* 33 15–29. 10.1016/j.molcel.2008.11.025 19150424

[B41] Fu N. Y. Sukumaran S. K. Yu V. C. (2007). Inhibition of ubiquitin-mediated degradation of MOAP-1 by apoptotic stimuli promotes Bax function in mitochondria. *Proc. Natl. Acad. Sci. U.S.A.* 104 10051–10056. 10.1073/pnas.0700007104 17535899 PMC1877986

[B42] Fujise K. Zhang D. Liu J. Yeh E. T. (2000). Regulation of apoptosis and cell cycle progression by MCL1. Differential role of proliferating cell nuclear antigen. *J. Biol. Chem.* 275 39458–39465. 10.1074/jbc.m006626200 10978339

[B43] Gavin M. A. Rasmussen J. P. Fontenot J. D. Vasta V. Manganiello V. C. Beavo J. A. (2007). Foxp3-dependent programme of regulatory T-cell differentiation. *Nature* 445 771–775. 10.1038/nature05543 17220874

[B44] Gendrin C. Vornhagen J. Ngo L. Whidbey C. Boldenow E. Santana-Ufret V. (2015). Mast cell degranulation by a hemolytic lipid toxin decreases GBS colonization and infection. *Sci. Adv.* 1:e1400225. 10.1126/sciadv.1400225 26425734 PMC4584422

[B45] Germain M. Nguyen A. P. Le Grand J. N. Arbour N. Vanderluit J. L. Park D. S. (2011). MCL-1 is a stress sensor that regulates autophagy in a developmentally regulated manner. *EMBO J.* 30 395–407. 10.1038/emboj.2010.327 21139567 PMC3025469

[B46] Gibson L. Holmgreen S. P. Huang D. C. Bernard O. Copeland N. G. Jenkins N. A. (1996). bcl-w, a novel member of the bcl-2 family, promotes cell survival. *Oncogene* 13 665–675. 8761287

[B47] Glaser S. P. Lee E. F. Trounson E. Bouillet P. Wei A. Fairlie W. D. (2012). Anti-apoptotic mcl-1 is essential for the development and sustained growth of acute myeloid leukemia. *Genes Dev.* 26 120–125. 10.1101/gad.182980.111 22279045 PMC3273836

[B48] Grabow S. Delbridge A. R. Valente L. J. Strasser A. (2014). MCL-1 but not BCL-XL is critical for the development and sustained expansion of thymic lymphoma in p53-deficient mice. *Blood* 124 3939–3946. 10.1182/blood-2014-09-601567 25368374

[B49] Grabow S. Kelly G. L. Delbridge A. R. D. Kelly P. N. Bouillet P. Adams J. M. (2016). Critical B-lymphoid cell intrinsic role of endogenous MCL-1 in c-MYC-induced lymphomagenesis. *Cell Death Dis.* 7 1–8. 10.1109/tmag.2013.2278570 PMC4823944 26962682

[B50] Gratiot-Deans J. Ding L. Turka L. A. Nuñez G. (1993). bcl-2 proto-oncogene expression during human T cell development. Evidence for biphasic regulation. *J. Immunol.* 151 83–91. 8326141

[B51] Gratiot-Deans J. Merino R. Nuñez G. Turka L. A. (1994). Bcl-2 expression during T-cell development: early loss and late return occur at specific stages of commitment to differentiation and survival. *Proc. Natl. Acad. Sci. U.S.A.* 91 10685–10689. 10.1073/pnas.91.22.10685 7938012 PMC45086

[B52] Green D. R. (2019). The coming decade of cell death research: five riddles. *Cell* 177 1094–1107. 10.1016/j.cell.2019.04.024 31100266 PMC6534278

[B53] Grillot D. A. Merino R. Núñez G. (1995). Bcl-XL displays restricted distribution during T cell development and inhibits multiple forms of apoptosis but not clonal deletion in transgenic mice. *J. Exp. Med.* 182 1973–1983. 10.1084/jem.182.6.1973 7500043 PMC2192263

[B54] Gui J. Hu Z. Tsai C.-Y. Ma T. Song Y. Morales A. (2015). MCL1 enhances the survival of CD8 + memory T cells after viral infection. *J. Virol.* 89 2405–2414. 10.1128/jvi.02480-14 25505074 PMC4338882

[B55] Guillerey C. Huntington N. D. Smyth M. J. (2016). Targeting natural killer cells in cancer immunotherapy. *Nat. Immunol.* 17 1025–1036. 27540992 10.1038/ni.3518

[B56] Han J. Flemington C. Houghton A. B. Gu Z. Zambetti G. P. Lutz R. J. (2001). Expression of bbc3, a pro-apoptotic BH3-only gene, is regulated by diverse cell death and survival signals. *Proc. Natl. Acad. Sci. U.S.A.* 98 11318–11323. 10.1073/pnas.201208798 11572983 PMC58727

[B57] Hébert J. M. McConnell S. K. (2000). Targeting of cre to the Foxg1 (BF-1) locus mediates loxP recombination in the telencephalon and other developing head structures. *Dev. Biol.* 222 296–306. 10.1006/dbio.2000.9732 10837119

[B58] Hirsova P. Bohm F. Dohnalkova E. Nozickova B. Heikenwalder M. Gores G. J. (2020). Hepatocyte apoptosis is tumor promoting in murine nonalcoholic steatohepatitis. *Cell Death Dis.* 11:80. 10.1038/s41419-020-2283-9 PMC6997423 32015322

[B59] Hirsova P. Guicciardi M. E. Gores G. J. (2017). Proapoptotic signaling induced by deletion of receptor-interacting kinase 1 and TNF receptor-associated factor 2 results in liver carcinogenesis. *Hepatology* 66 983–985. 10.1002/hep.29272 28520112 PMC5570646

[B60] Huntington N. D. Puthalakath H. Gunn P. Naik E. Michalak E. M. Smyth M. J. (2007). Interleukin 15-mediated survival of natural killer cells is determined by interactions among Bim, Noxa and Mcl-1. *Nat. Immunol.* 8 856–863. 10.1038/ni1487 17618288 PMC2951739

[B61] Inohara N. Ding L. Chen S. Núñez G. (1997). harakiri, a novel regulator of cell death, encodes a protein that activates apoptosis and interacts selectively with survival-promoting proteins Bcl-2 and Bcl-X(L). *Embo J.* 16 1686–1694. 10.1093/emboj/16.7.1686 9130713 PMC1169772

[B62] Inuzuka H. Shaik S. Onoyama I. Gao D. Tseng A. Maser R. S. (2011). SCF(FBW7) regulates cellular apoptosis by targeting MCL1 for ubiquitylation and destruction. *Nature* 471 104–109. 10.1038/nature09732 21368833 PMC3076007

[B63] Jain R. Sheridan J. M. Policheni A. Heinlein M. Luke C. (2018). A critical epithelial survival axis regulated by MCL-1 maintains thymic function in mice. *Blood* 130 2504–2515. 10.1182/blood-2017-03-771576 28972012 PMC6207344

[B64] Jamil S. Sobouti R. Hojabrpour P. Raj M. Kast J. Duronio V. (2005). A proteolytic fragment of Mcl-1 exhibits nuclear localization and regulates cell growth by interaction with Cdk1. *Biochem. J.* 387 659–667. 10.1042/bj20041596 15554878 PMC1134995

[B65] Josefsson E. C. James C. Henley K. J. Debrincat M. A. Rogers K. L. Dowling M. R. (2011). Megakaryocytes possess a functional intrinsic apoptosis pathway that must be restrained to survive and produce platelets. *J. Exp. Med.* 208 2017–2031. 10.1084/jem.20110750 21911424 PMC3182050

[B66] Kahraman A. Mott J. L. Bronk S. F. Werneburg N. W. Barreyro F. J. Guicciardi M. E. (2009). Overexpression of mcl-1 attenuates liver injury and fibrosis in the bile duct-ligated mouse. *Dig. Dis. Sci.* 54 1908–1917. 10.1007/s10620-008-0583-5 19051025 PMC2879585

[B67] Kale J. Osterlund E. J. Andrews D. W. (2018). BCL-2 family proteins: changing partners in the dance towards death. *Cell Death Differ.* 25 65–80. 10.1038/cdd.2017.186 29149100 PMC5729540

[B68] Kaufmann S. H. Karp J. E. Svingen P. A. Krajewski S. Burke P. J. Gore S. D. (1998). Elevated expression of the apoptotic regulator Mcl-1 at the time of leukemic relapse. *Blood* 91 991–1000. 10.1182/blood.v91.3.991.991_991_1000 9446661

[B69] Ke F. Voss A. Kerr J. B. O’Reilly L. A. Tai L. Echeverry N. (2012). BCL-2 family member BOK is widely expressed but its loss has only minimal impact in mice. *Cell Death Differ.* 19 915–925. 10.1038/cdd.2011.210 22281706 PMC3354060

[B70] Kelly G. L. Grabow S. Glaser S. P. Fitzsimmons L. Aubrey B. J. Okamoto T. (2014). Targeting of MCL-1 kills MYC-driven mouse and human lymphomas even when they bear mutations in p53. *Genes Dev.* 28 58–70. 10.1101/gad.232009.113 24395247 PMC3894413

[B71] Kelly G. L. Strasser A. (2020). Toward Targeting Antiapoptotic MCL-1 for Cancer Therapy. *Ann. Rev. Cancer Biol.* 4 299–313. 10.1146/annurev-cancerbio-030419-033510

[B72] Kerr J. F. Wyllie A. H. Currie A. R. (1972). Apoptosis: a basic biological phenomenon with wide-ranging implications in tissue kinetics. *Br. J. Cancer* 26 239–257. 10.1038/bjc.1972.33 4561027 PMC2008650

[B73] Kiefer M. C. Brauer M. J. Powers V. C. Wu J. J. Umansky S. R. Tomei L. D. (1995). Modulation of apoptosis by the widely distributed Bcl-2 homologue Bak. *Nature* 374 736–739. 10.1038/374736a0 7715731

[B74] Kim E. H. Neldner B. Gui J. Craig R. W. Suresh M. (2016). Mcl-1 regulates effector and memory CD8 T-cell differentiation during acute viral infection. *Virology* 490 75–82. 10.1016/j.virol.2016.01.008 26855329 PMC4769930

[B75] Kisanuki Y. Y. Hammer R. E. Miyazaki J. Williams S. C. Richardson J. A. Yanagisawa M. (2001). Tie2-Cre transgenic mice: a new model for endothelial cell-lineage analysis in vivo. *Dev. Biol.* 230 230–242. 10.1006/dbio.2000.0106 11161575

[B76] Kluck R. M. Bossy-Wetzel E. Green D. R. Newmeyer D. D. (1997). The release of cytochrome c from mitochondria: a primary site for Bcl-2 regulation of apoptosis. *Science* 275 1132–1136. 10.1126/science.275.5303.1132 9027315

[B77] Kotschy A. Szlavik Z. Murray J. Davidson J. Maragno A. L. Le Toumelin-Braizat G. (2016). The MCL1 inhibitor S63845 is tolerable and effective in diverse cancer models. *Nature* 538 477–482. 27760111 10.1038/nature19830

[B78] Kozopas K. M. Yang T. Buchan H. L. Zhou P. Craig R. W. (1993). MCL1, a gene expressed in programmed myeloid cell differentiation, has sequence similarity to BCL2. *Proc. Natl. Acad. Sci. U.S.A.* 90 3516–3520. 10.1073/pnas.90.8.3516 7682708 PMC46331

[B79] Krueger A. von Boehmer H. (2007). Identification of a T lineage-committed progenitor in adult blood. *Immunity* 26 105–116. 10.1016/j.immuni.2006.12.004 17222572 PMC1828638

[B80] Kuhn R. Schwenk F. Aguet M. Rajewsky K. (1995). Inducible gene targeting in mice. *Science* 269 1427–1429. 10.1126/science.7660125 7660125

[B81] Kumar B. V. Connors T. J. Farber D. L. (2018). Human T Cell Development, Localization, and Function throughout Life. *Immunity* 48 202–213. 10.1016/j.immuni.2018.01.007 29466753 PMC5826622

[B82] Kuwana T. Bouchier-Hayes L. Chipuk J. E. Bonzon C. Sullivan B. A. Green D. R. (2005). BH3 domains of BH3-only proteins differentially regulate Bax-mediated mitochondrial membrane permeabilization both directly and indirectly. *Mol Cell* 17 525–535. 10.1016/j.molcel.2005.02.003 15721256

[B83] Kwon K. Hutter C. Sun Q. Bilic I. Cobaleda C. Malin S. (2008). Instructive role of the transcription factor E2A in early B lymphopoiesis and germinal center B cell development. *Immunity* 28 751–762. 10.1016/j.immuni.2008.04.014 18538592

[B84] Lavau C. Luo R. T. Du C. Thirman M. J. (2000). Retrovirus-mediated gene transfer of MLL-ELL transforms primary myeloid progenitors and causes acute myeloid leukemias in mice. *Proc. Natl. Acad. Sci. U.S.A.* 97 10984–10989. 10.1073/pnas.190167297 10995463 PMC27135

[B85] Lee P. P. Fitzpatrick D. R. Beard C. Jessup H. K. Lehar S. Makar K. W. (2001). A critical role for Dnmt1 and DNA methylation in T cell development, function, and survival. *Immunity* 15 763–774. 10.1016/s1074-7613(01)00227-8 11728338

[B86] Letai A. Bassik M. C. Walensky L. D. Sorcinelli M. D. Weiler S. Korsmeyer S. J. (2002). Distinct BH3 domains either sensitize or activate mitochondrial apoptosis, serving as prototype cancer therapeutics. *Cancer Cell* 2 183–192. 10.1016/s1535-6108(02)00127-7 12242151

[B87] Leveson-Gower D. B. Sega E. I. Kalesnikoff J. Florek M. Pan Y. Pierini A. (2013). Mast cells suppress murine GVHD in a mechanism independent of CD4+CD25+ regulatory T cells. *Blood* 122 3659–3665. 10.1182/blood-2013-08-519157 24030387 PMC3837515

[B88] Lewandoski M. Wassarman K. M. Martin G. R. (1997). Zp3-cre, a transgenic mouse line for the activation or inactivation of loxP-flanked target genes specifically in the female germ line. *Curr. Biol. CB* 7 148–151. 10.1016/s0960-9822(06)00059-5 9016703

[B89] Li H. Wang J. Wilhelmsson H. Hansson A. Thoren P. Duffy J. (2000). Genetic modification of survival in tissue-specific knockout mice with mitochondrial cardiomyopathy. *Proc. Natl. Acad. Sci. U.S.A.* 97 3467–3472. 10.1073/pnas.97.7.3467 10737799 PMC16263

[B90] Lilla J. N. Chen C. G. Mukai K. BenBarak M. J. Franco C. B. Kalesnikoff J. (2011). Reduced mast cell and basophil numbers and function in Cpa3-Cre; Mcl-1 fl/fl mice. *Blood* 118 6930–6938. 10.1182/blood-2011-03-343962 22001390 PMC3245213

[B91] Lin E. Y. Orlofsky A. Berger M. S. Prystowsky M. B. (1993). Characterization of A1, a novel hemopoietic-specific early-response gene with sequence similarity to bcl-2. *J. Immunol.* 151 1979–1988. 8345191

[B92] Liu Y. J. (2005). IPC: professional type 1 interferon-producing cells and plasmacytoid dendritic cell precursors. *Annu. Rev. Immunol.* 23 275–306. 10.1146/annurev.immunol.23.021704.115633 15771572

[B93] Luo Y. Meyer N. Jiao Q. Scheffel J. Zimmermann C. Metz M. (2019). Chymase-Cre; Mcl-1fl/fl mice exhibit reduced numbers of mucosal mast cells. *Front. Immunol.* 10:2399. 10.3389/fimmu.2019.02399 31681290 PMC6803453

[B94] MacLennan I. C. (1994). Germinal centers. *Ann. Rev. Immunol.* 12 117–139. 8011279 10.1146/annurev.iy.12.040194.001001

[B95] Magiera M. M. Mora S. Mojsa B. Robbins I. Lassot I. Desagher S. (2013). Trim17-mediated ubiquitination and degradation of Mcl-1 initiate apoptosis in neurons. *Cell Death Differ.* 20 281–292. 10.1038/cdd.2012.124 22976837 PMC3554334

[B96] Marichal T. Starkl P. Reber L. L. Kalesnikoff J. Oettgen H. C. Tsai M. (2013). A beneficial role for immunoglobulin E in host defense against honeybee venom. *Immunity* 39 963–975. 10.1016/j.immuni.2013.10.005 24210352 PMC4164235

[B97] Mason K. D. Carpinelli M. R. Fletcher J. I. Collinge J. E. Hilton A. A. Ellis S. (2007). Programmed anuclear cell death delimits platelet life span. *Cell* 128 1173–1186. 10.1016/j.cell.2007.01.037 17382885

[B98] Merino D. Kelly G. L. Lessene G. Wei A. H. Roberts A. W. Strasser A. (2018). BH3-mimetic drugs: blazing the trail for new cancer medicines. *Cancer Cell* 34 879–891. 10.1016/j.ccell.2018.11.004 30537511

[B99] Mills J. R. Hippo Y. Robert F. Chen S. M. Malina A. Lin C. J. (2008). mTORC1 promotes survival through translational control of Mcl-1. *Proc. Natl. Acad. Sci. U.S.A.* 105 10853–10858. 10.1073/pnas.0804821105 18664580 PMC2504845

[B100] Min B. (2011). Deleting Mcl-1 in mast cells: getting 2 birds with 1 stone. *Blood* 118 6729–6730. 10.1182/blood-2011-10-386565 22194394

[B101] Mojsa B. Lassot I. Desagher S. (2014). Mcl-1 ubiquitination: unique regulation of an essential survival protein. *Cells* 3 418–437. 10.3390/cells3020418 24814761 PMC4092850

[B102] Nakano K. Vousden K. H. (2001). PUMA, a novel proapoptotic gene, is induced by p53. *Mol. Cell* 7 683–694. 10.1016/s1097-2765(01)00214-3 11463392

[B103] Nakayama K. Nakayama K. Negishi I. Kuida K. Shinkai Y. Louie M. C. (1993). Disappearance of the lymphoid system in Bcl-2 homozygous mutant chimeric mice. *Science* 261 1584–1588. 10.1126/science.8372353 8372353

[B104] Narni-Mancinelli E. Chaix J. Fenis A. Kerdiles Y. M. Yessaad N. Reynders A. (2011). Fate mapping analysis of lymphoid cells expressing the NKp46 cell surface receptor. *Proc. Natl. Acad. Sci. U.S.A.* 108 18324–18329. 10.1073/pnas.1112064108 22021440 PMC3215049

[B105] O’Connor L. Strasser A. O’Reilly L. A. Hausmann G. Adams J. M. Cory S. (1998). Bim: a novel member of the Bcl-2 family that promotes apoptosis. *Embo J.* 17 384–395. 10.1093/emboj/17.2.384 9430630 PMC1170389

[B106] Oda E. Ohki R. Murasawa H. Nemoto J. Shibue T. Yamashita T. (2000). Noxa, a BH3-only member of the Bcl-2 family and candidate mediator of p53-induced apoptosis. *Science* 288 1053–1058. 10.1126/science.288.5468.1053 10807576

[B107] Ogilvy S. Metcalf D. Gibson L. Bath M. L. Harris A. W. Adams J. M. (1999). Promoter elements of vav drive transgene expression in vivo throughout the hematopoietic compartment. *Blood* 94 1855–1863. 10.1182/blood.v94.6.1855 10477714

[B108] Okamoto T. Coultas L. Metcalf D. Van Delft M. F. Glaser S. P. Takiguchi M. (2014). Enhanced stability of Mcl1, a prosurvival Bcl2 relative, blunts stress-induced apoptosis, causes male sterility, and promotes tumorigenesis. *Proc. Natl. Acad. Sci. U.S.A.* 111 261–266. 10.1073/pnas.1321259110 24363325 PMC3890801

[B109] Oltvai Z. N. Milliman C. L. Korsmeyer S. J. (1993). Bcl-2 heterodimerizes in vivo with a conserved homolog, Bax, that accelerates programmed cell death. *Cell* 74 609–619. 10.1016/0092-8674(93)90509-o 8358790

[B110] Omari S. Waters M. Naranian T. Kim K. Perumalsamy A. L. Chi M. (2015). Mcl-1 is a key regulator of the ovarian reserve. *Cell Death Dis.* 6:e1755. 10.1038/cddis.2015.95 25950485 PMC4669721

[B111] O’Neill K. L. Huang K. Zhang J. Chen Y. Luo X. (2016). Inactivation of prosurvival Bcl-2 proteins activates Bax/Bak through the outer mitochondrial membrane. *Genes Dev.* 30 973–988. 10.1101/gad.276725.115 27056669 PMC4840302

[B112] Opferman J. T. Iwasaki H. Ong C. C. Suh H. Mizuno S. I. Akashi K. (2005). Obligate role of anti-apoptotic MCL-1 in the survival of hematopoietic stem cells. *Science* 307 1101–1104. 10.1126/science.1106114 15718471

[B113] Opferman J. T. Letai A. Beard C. Sorcinelli M. D. Ong C. C. Korsmeyer S. J. (2003). Development and maintenance of B and T lymphocytes requires antiapoptotic MCL-1. *Nature* 426 671–676. 10.1038/nature02067 14668867

[B114] Passegué E. Wagner E. F. Weissman I. L. (2004). JunB deficiency leads to a myeloproliferative disorder arising from hematopoietic stem cells. *Cell* 119 431–443. 10.1016/j.cell.2004.10.010 15507213

[B115] Peperzak V. Vikström I. Walker J. Glaser S. P. Lepage M. Coquery C. M. (2013). Mcl-1 is essential for the survival of plasma cells. *Nat. Immunol.* 14 290–297. 10.1038/ni.2527 23377201 PMC4041127

[B116] Perciavalle R. M. Opferman J. T. (2014). Delving Deeper: MCL-1’s Contributions to Normal and Cancer Biology. *Bone* 23 1–7. 10.1016/j.tcb.2012.08.011 PMC3532576 23026029

[B117] Perciavalle R. M. Stewart D. P. Koss B. Lynch J. Milasta S. Bathina M. (2012). Anti-apoptotic MCL-1 localizes to the mitochondrial matrix and couples mitochondrial fusion to respiration. *Nat. Cell Biol.* 14 575–583. 10.1038/ncb2488 22544066 PMC3401947

[B118] Pierson W. Cauwe B. Policheni A. Schlenner S. M. Franckaert D. Berges J. (2013). Antiapoptotic Mcl-1 is critical for the survival and niche-filling capacity of Foxp3^+^ regulatory T cells. *Nat. Immunol.* 14 959–965. 10.1038/ni.2649 23852275 PMC4128388

[B119] Postic C. Magnuson M. A. (2000). DNA excision in liver by an albumin-Cre transgene occurs progressively with age. *Genesis (New York, NY : 2000)* 26 149–150. 10.1002/(sici)1526-968x(200002)26:2<149::aid-gene16>3.0.co;2-v 10686614

[B120] Premsrirut P. K. Dow L. E. Kim S. Y. Camiolo M. Malone C. D. Miething C. (2011). A rapid and scalable system for studying gene function in mice using conditional RNA interference. *Cell* 145 145–158. 10.1016/j.cell.2011.03.012 21458673 PMC3244080

[B121] Print C. G. Loveland K. L. Gibson L. Meehan T. Stylianou A. Wreford N. (1998). Apoptosis regulator Bcl-w is essential for spermatogenesis but appears otherwise redundant. *Proc. Natl. Acad. Sci. U.S.A.* 95:12424. 10.1073/pnas.95.21.12424 9770502 PMC22847

[B122] Puthalakath H. Villunger A. O’Reilly L. A. Beaumont J. G. Coultas L. Cheney R. E. (2001). Bmf: a proapoptotic BH3-only protein regulated by interaction with the myosin V actin motor complex, activated by anoikis. *Science* 293 1829–1832. 10.1126/science.1062257 11546872

[B123] Reber L. L. Marichal T. Mukai K. Kita Y. Tokuoka S. M. Roers A. (2013). Selective ablation of mast cells or basophils reduces peanut-induced anaphylaxis in mice. *J. Allergy Clin. Immunol.* 132 881–888.e881–811. 23915716 10.1016/j.jaci.2013.06.008 PMC3794715

[B124] Rechsteiner M. Rogers S. W. (1996). PEST sequences and regulation by proteolysis. *Trends Biochem. Sci.* 21 267–271. 10.1016/s0968-0004(96)10031-1 8755249

[B125] Reynolds J. E. Yang T. Qian L. Jenkinson J. D. Zhou P. Eastman A. (1994). Mcl-1, a member of the Bcl-2 family, delays apoptosis induced by c-Myc overexpression in Chinese hamster ovary cells. *Cancer Res.* 54 6348–6352. 7987827

[B126] Rickert R. C. Roes J. Rajewsky K. (1997). B lymphocyte-specific, Cre-mediated mutagenesis in mice. *Nucleic Acids Res.* 25 1317–1318. 10.1093/nar/25.6.1317 9092650 PMC146582

[B127] Rinkenberger J. L. Horning S. Klocke B. Roth K. Korsmeyer S. J. (2000). Mcl-1 deficiency results in peri-implantation embryonic lethality. *Genes Dev.* 14 23–27. 10640272 PMC316347

[B128] Roberts A. W. Seymour J. F. Brown J. R. Wierda W. G. Kipps T. J. Khaw S. L. (2012). Substantial susceptibility of chronic lymphocytic leukemia to BCL2 inhibition: results of a phase I study of navitoclax in patients with relapsed or refractory disease. *J. Clin. Oncol.* 30 488–496. 10.1200/jco.2011.34.7898 22184378 PMC4979082

[B129] Ross A. J. Waymire K. G. Moss J. E. Parlow A. F. Skinner M. K. Russell L. D. (1998). Testicular degeneration in Bclw-deficient mice. *Nat. Genet.* 18 251–256. 10.1038/ng0398-251 9500547

[B130] Rubtsov Y. P. Rasmussen J. P. Chi E. Y. Fontenot J. Castelli L. Ye X. (2008). Regulatory T cell-derived interleukin-10 limits inflammation at environmental interfaces. *Immunity* 28 546–558. 10.1016/j.immuni.2008.02.017 18387831

[B131] Sale M. J. Minihane E. Monks N. R. Gilley R. Richards F. M. Schifferli K. P. (2019). Targeting melanoma’s MCL1 bias unleashes the apoptotic potential of BRAF and ERK1/2 pathway inhibitors. *Nat. Commun.* 10:5167. 10.1038/s41467-019-12409-w PMC6856071 31727888

[B132] Sathe P. Delconte R. B. Souza-Fonseca-Guimaraes F. Seillet C. Chopin M. Vandenberg C. J. (2014). Innate immunodeficiency following genetic ablation of Mcl1 in natural killer cells. *Nat. Commun.* 5:4539. 10.1038/ncomms5539 25119382

[B133] Schenk R. L. Tuzlak S. Carrington E. M. Zhan Y. Heinzel S. Teh C. E. (2017). Characterisation of mice lacking all functional isoforms of the pro-survival BCL-2 family member A1 reveals minor defects in the haematopoietic compartment. *Cell Death Differ.* 24 534–545. 10.1038/cdd.2016.156 28085150 PMC5344213

[B134] Schlenner S. M. Madan V. Busch K. Tietz A. Läufle C. Costa C. (2010). Fate mapping reveals separate origins of T cells and myeloid lineages in the thymus. *Immunity* 32 426–436. 10.1016/j.immuni.2010.03.005 20303297

[B135] Schwickart M. Huang X. Lill J. R. Liu J. Ferrando R. French D. M. (2010). Deubiquitinase USP9X stabilizes MCL1 and promotes tumour cell survival. *Nature* 463 103–107. 10.1038/nature08646 20023629

[B136] Senichkin V. V. Streletskaia A. Y. Gorbunova A. S. Zhivotovsky B. Kopeina G. S. (2020). Saga of Mcl-1: regulation from transcription to degradation. *Cell Death Differ.* 27 405–419. 10.1038/s41418-019-0486-3 31907390 PMC7206148

[B137] Sieghart W. Losert D. Strommer S. Cejka D. Schmid K. Rasoul-Rockenschaub S. (2006). Mcl-1 overexpression in hepatocellular carcinoma: a potential target for antisense therapy. *J. Hepatol.* 44 151–157. 10.1016/j.jhep.2005.09.010 16289418

[B138] Simmons M. J. Fan G. Zong W. X. Degenhardt K. White E. Gélinas C. (2008). Bfl-1/A1 functions, similar to Mcl-1, as a selective tBid and Bak antagonist. *Oncogene* 27 1421–1428. 10.1038/sj.onc.1210771 17724464 PMC2880719

[B139] Singh R. Letai A. Sarosiek K. (2019). Regulation of apoptosis in health and disease: the balancing act of BCL-2 family proteins. *Nat. Rev. Mol. Cell Biol.* 20 175–193. 10.1038/s41580-018-0089-8 30655609 PMC7325303

[B140] Sohal D. S. Nghiem M. Crackower M. A. Witt S. A. Kimball T. R. Tymitz K. M. (2001). Temporally regulated and tissue-specific gene manipulations in the adult and embryonic heart using a tamoxifen-inducible Cre protein. *Circ. Res.* 89 20–25. 10.1161/hh1301.092687 11440973

[B141] Spinner S. Crispatzu G. Yi J. H. Munkhbaatar E. Mayer P. Höckendorf U. (2016). Re-activation of mitochondrial apoptosis inhibits T-cell lymphoma survival and treatment resistance. *Leukemia* 30 1520–1530. 10.1038/leu.2016.49 27055871

[B142] Steimer D. A. Boyd K. Takeuchi O. Fisher J. K. Zambetti G. P. Opferman J. T. (2009). Selective roles for antiapoptotic MCL-1 during granulocyte development and macrophage effector function. *Blood* 113 2805–2815. 10.1182/blood-2008-05-159145 19064728 PMC2661864

[B143] Steinman R. M. (2012). Decisions about dendritic cells: past, present, and future. *Annu. Rev. Immunol.* 30 1–22. 10.1146/annurev-immunol-100311-102839 22136168

[B144] Strasser A. Harris A. W. Bath M. L. Cory S. (1990). Novel primitive lymphoid tumours induced in transgenic mice by cooperation between myc and bcl-2. *Nature* 348 331–333. 10.1038/348331a0 2250704

[B145] Strasser A. Whittingham S. Vaux D. L. Bath M. L. Adams J. M. Cory S. (1991). Enforced BCL2 expression in B-lymphoid cells prolongs antibody responses and elicits autoimmune disease. *Proc. Natl. Acad. Sci. U.S.A.* 88:8661. 10.1073/pnas.88.19.8661 1924327 PMC52569

[B146] Szlavik Z. Csekei M. Paczal A. Szabo Z. B. Sipos S. Radics G. (2020). Discovery of S64315, a potent and selective Mcl-1 inhibitor. *J. Med. Chem.* 63 13762–13795. 33146521 10.1021/acs.jmedchem.0c01234

[B147] Tan K. O. Tan K. M. Chan S. L. Yee K. S. Bevort M. Ang K. C. (2001). MAP-1, a novel proapoptotic protein containing a BH3-like motif that associates with Bax through its Bcl-2 homology domains. *J. Biol. Chem.* 276 2802–2807. 10.1074/jbc.m008955200 11060313

[B148] Thomas L. W. Lam C. Edwards S. W. (2010). Mcl-1; the molecular regulation of protein function. *FEBS Lett.* 584 2981–2989. 20540941 10.1016/j.febslet.2010.05.061

[B149] Thomas R. L. Roberts D. J. Kubli D. A. Lee Y. Quinsay M. N. Owens J. B. (2013). Loss of MCL-1 leads to impaired autophagy and rapid development of heart failure. *Genes Dev.* 27 1365–1377. 10.1101/gad.215871.113 23788623 PMC3701192

[B150] Thorp E. Li Y. Bao L. Yao P. M. Kuriakose G. Rong J. (2009). Brief report: increased apoptosis in advanced atherosclerotic lesions of Apoe-/- mice lacking macrophage Bcl-2. *Arterioscler. Thromb. Vasc. Biol.* 29 169–172. 10.1161/atvbaha.108.176495 18988889 PMC2731712

[B151] Tripathi P. Koss B. Opferman J. T. Hildeman D. A. (2013). Mcl-1 antagonizes Bax/Bak to promote effector CD4+ and CD8 + T-cell responses. *Cell Death Differ.* 20 998–1007. 10.1038/cdd.2013.25 23558951 PMC3705594

[B152] Tron A. E. Belmonte M. A. Adam A. Aquila B. M. Boise L. H. Chiarparin E. (2018). Discovery of Mcl-1-specific inhibitor AZD5991 and preclinical activity in multiple myeloma and acute myeloid leukemia. *Nat. Commun.* 9:5341. 10.1038/s41467-018-07551-w PMC6297231 30559424

[B153] Vaux D. L. Cory S. Adams J. M. (1988). Bcl-2 gene promotes haemopoietic cell survival and cooperates with c-myc to immortalize pre-B cells. *Nature* 335 440–442. 10.1038/335440a0 3262202

[B154] Veis D. J. Sorenson C. M. Shutter J. R. Korsmeyer S. J. (1993). Bcl-2-deficient mice demonstrate fulminant lymphoid apoptosis, polycystic kidneys, and hypopigmented hair. *Cell* 75 229–240. 10.1016/0092-8674(93)80065-m 8402909

[B155] Viant C. Guia S. Hennessy R. J. Rautela J. Pham K. Bernat C. (2017). Cell cycle progression dictates the requirement for BCL2 in natural killer cell survival. *J. Exp. Med.* 214 491–510. 10.1084/jem.20160869 28057804 PMC5294858

[B156] Vick B. Weber A. Urbanik T. Maass T. Teufel A. Peter H. (2009). Knock-out of Myeloid cell leukemia-1 induces liver damage and increases apoptosis susceptibility of murine hepatocytes. *Hepatology* 49 627–636. 10.1002/hep.22664 19127517 PMC2753874

[B157] Vikstrom I. Carotta S. Lüthje K. Peperzak V. Jost P. J. Glaser S. (2010). Mcl-1 is essential for germinal center formation and B cell memory. *Science* 330 1095–1099. 10.1126/science.1191793 20929728 PMC2991396

[B158] Vikström I. B. Slomp A. Carrington E. M. Moesbergen L. M. Chang C. Kelly G. L. (2016). MCL-1 is required throughout B-cell development and its loss sensitizes specific B-cell subsets to inhibition of BCL-2 or BCL-XL. *Cell Death Dis.* 7 1–9. 10.1038/cddis.2016.237 PMC5108322 27560714

[B159] Wagner K. U. Claudio E. Rucker E. B. III Riedlinger G. Broussard C. Schwartzberg P. L. (2000). Conditional deletion of the Bcl-x gene from erythroid cells results in hemolytic anemia and profound splenomegaly. *Development* 127 4949–4958. 10.1242/dev.127.22.4949 11044408

[B160] Walton K. D. Wagner K.-U. Rucker E. B. Shillingford J. M. Miyoshi K. Hennighausen L. (2001). Conditional deletion of the bcl-x gene from mouse mammary epithelium results in accelerated apoptosis during involution but does not compromise cell function during lactation. *Mech. Dev.* 109 281–293. 10.1016/s0925-4773(01)00549-4 11731240

[B161] Wang J. M. Chao J. R. Chen W. Kuo M. L. Yen J. J. Yang-Yen H. F. (1999). The antiapoptotic gene mcl-1 is up-regulated by the phosphatidylinositol 3-kinase/Akt signaling pathway through a transcription factor complex containing CREB. *Mol. Cell. Biol.* 19 6195–6206. 10.1128/mcb.19.9.6195 10454566 PMC84561

[B162] Wang K. Yin X. M. Chao D. T. Milliman C. L. Korsmeyer S. J. (1996). BID: a novel BH3 domain-only death agonist. *Genes Dev.* 10 2859–2869. 10.1101/gad.10.22.2859 8918887

[B163] Wang Q. Lepus C. M. Raghu H. Reber L. L. Tsai M. M. Wong H. H. (2019). Ige-mediated mast cell activation promotes inflammation and cartilage destruction in osteoarthritis. *eLife* 8 1–23. 10.1016/j.joca.2017.10.005 31084709 PMC6516833

[B164] Wang X. Bathina M. Lynch J. Koss B. Calabrese C. Frase S. (2013). Deletion of MCL-1 causes lethal cardiac failure and mitochondrial dysfunction. *Genes Dev.* 27 1351–1364. 10.1101/gad.215855.113 23788622 PMC3701191

[B165] Wang Y. Nakayama M. Pitulescu M. E. Schmidt T. S. Bochenek M. L. Sakakibara A. (2010). Ephrin-B2 controls VEGF-induced angiogenesis and lymphangiogenesis. *Nature* 465 483–486. 10.1038/nature09002 20445537

[B166] Watson E. C. Whitehead L. Adams R. H. Dewson G. Coultas L. (2016). Endothelial cell survival during angiogenesis requires the pro-survival protein MCL1. *Cell Death Differ.* 23 1371–1379. 10.1038/cdd.2016.20 26943318 PMC4947668

[B167] Weber A. Boger R. Vick B. Urbanik T. Haybaeck J. Zoller S. (2010). Hepatocyte-specific deletion of the antiapoptotic protein myeloid cell leukemia-1 triggers proliferation and hepatocarcinogenesis in mice. *Hepatology* 51 1226–1236. 10.1002/hep.23479 20099303 PMC2936921

[B168] Wei A. H. Roberts A. W. Spencer A. Rosenberg A. S. Siegel D. Walter R. B. (2020). Targeting MCL-1 in hematologic malignancies: rationale and progress. *Blood Rev.* 44:100672. 10.1016/j.blre.2020.100672 32204955 PMC7442684

[B169] Wei G. Margolin A. A. Haery L. Brown E. Cucolo L. Julian B. (2012). Chemical genomics identifies small-molecule MCL1 repressors and BCL-xL as a predictor of MCL1 dependency. *Cancer Cell* 21 547–562. 10.1016/j.ccr.2012.02.028 22516262 PMC3685408

[B170] Wensveen F. M. Derks I. A. van Gisbergen K. P. de Bruin A. M. Meijers J. C. Yigittop H. (2012). BH3-only protein Noxa regulates apoptosis in activated B cells and controls high-affinity antibody formation. *Blood* 119 1440–1449. 10.1182/blood-2011-09-378877 22144184

[B171] Wensveen F. M. Slinger E. van Attekum M. H. Brink R. Eldering E. (2016). Antigen-affinity controls pre-germinal center B cell selection by promoting Mcl-1 induction through BAFF receptor signaling. *Sci. Rep.* 6:35673. 10.1038/srep35673 PMC5071843 27762293

[B172] Wensveen F. M. van Gisbergen K. P. Derks I. A. Gerlach C. Schumacher T. N. van Lier R. A. (2010). Apoptosis threshold set by Noxa and Mcl-1 after T cell activation regulates competitive selection of high-affinity clones. *Immunity* 32 754–765. 10.1016/j.immuni.2010.06.005 20620942

[B173] Willis S. N. Chen L. Dewson G. Wei A. Naik E. Fletcher J. I. (2005). Proapoptotic Bak is sequestered by Mcl-1 and Bcl-xL, but not Bcl-2, until displaced by BH3-only proteins. *Genes Dev.* 19 1294–1305. 10.1101/gad.1304105 15901672 PMC1142553

[B174] Willis S. N. Fletcher J. I. Kaufmann T. van Delft M. F. Chen L. Czabotar P. E. (2007). Apoptosis initiated when BH3 ligands engage multiple Bcl-2 homologs, not Bax or Bak. *Science* 315 856–859. 10.1126/science.1133289 17289999

[B175] Wintermantel T. M. Mayer A. K. Schütz G. Greiner E. F. (2002). Targeting mammary epithelial cells using a bacterial artificial chromosome. *Genesis (New York, NY : 2000)* 33 125–130. 10.1002/gene.10097 12124945

[B176] Xiang W. Yang C.-Y. Bai L. (2018). MCL-1 inhibition in cancer treatment. *Onco. Targets Ther.* 11 7301–7314. 10.2147/ott.s146228 30425521 PMC6205821

[B177] Xiang Z. Luo H. Payton J. E. Cain J. Ley T. J. Opferman J. T. (2010). Mcl1 haploinsufficiency protects mice from Myc-induced acute myeloid leukemia. *J. Clin. Invest.* 120 2109–2118. 10.1172/jci39964 20484815 PMC2877934

[B178] Yang C. Y. Lin N. H. Lee J. M. Huang C. Y. Min H. J. Yen J. J. (2009). Promoter knock-in mutations reveal a role of Mcl-1 in thymocyte-positive selection and tissue or cell lineage-specific regulation of Mcl-1 expression. *J. Immunol.* 182 2959–2968. 10.4049/jimmunol.0803550 19234191

[B179] Yang E. Zha J. Jockel J. Boise L. H. Thompson C. B. Korsmeyer S. J. (1995). Bad, a heterodimeric partner for Bcl-XL and Bcl-2, displaces Bax and promotes cell death. *Cell* 80 285–291. 10.1016/0092-8674(95)90411-5 7834748

[B180] Zack T. I. Schumacher S. E. Carter S. L. Cherniack A. D. Saksena G. Tabak B. (2013). Pan-cancer patterns of somatic copy number alteration. *Nat. Genet.* 45 1134–1140. 24071852 10.1038/ng.2760 PMC3966983

[B181] Zhang N. He Y.-W. (2005). The antiapoptotic protein Bcl-xL is dispensable for the development of effector and memory T lymphocytes. *J. Immunol.* 174:6967. 10.4049/jimmunol.174.11.6967 15905539

[B182] Zhong Q. Gao W. Du F. Wang X. (2005). Mule/ARF-BP1, a BH3-only E3 ubiquitin ligase, catalyzes the polyubiquitination of Mcl-1 and regulates apoptosis. *Cell* 121 1085–1095. 10.1016/j.cell.2005.06.009 15989957

[B183] Zhou P. Levy N. B. Xie H. Qian L. Lee C. Y. G. Gascoyne R. D. (2001). MCL1 transgenic mice exhibit a high incidence of B-cell lymphoma manifested as a spectrum of histologic subtypes. *Blood* 97 3902–3909. 10.1182/blood.v97.12.3902 11389033

[B184] Zhou P. Qian L. Bieszczad C. K. Noelle R. Binder M. Levy N. B. (1998). Mcl-1 in transgenic mice promotes survival in a spectrum of hematopoietic cell types and immortalization in the myeloid lineage. *Blood* 92 3226–3239. 10.1182/blood.v92.9.3226 9787159

[B185] Zhou P. Qian L. Kozopas K. M. Craig R. W. (1997). Mcl-1, a Bcl-2 family member, delays the death of hematopoietic cells under a variety of apoptosis-inducing conditions. *Blood* 89 630–643. 10.1182/blood.v89.2.630 9002967

[B186] Zuklys S. Gill J. Keller M. P. Hauri-Hohl M. Zhanybekova S. Balciunaite G. (2009). Stabilized beta-catenin in thymic epithelial cells blocks thymus development and function. *J. Immunol.* 182 2997–3007. 10.4049/jimmunol.0713723 19234195

